# Identification of RCC Subtype-Specific microRNAs–Meta-Analysis of High-Throughput RCC Tumor microRNA Expression Data

**DOI:** 10.3390/cancers13030548

**Published:** 2021-02-01

**Authors:** Arkadiusz Kajdasz, Weronika Majer, Katarzyna Kluzek, Jacek Sobkowiak, Tomasz Milecki, Natalia Derebecka, Zbigniew Kwias, Hans A. R. Bluyssen, Joanna Wesoly

**Affiliations:** 1 Laboratory of Human Molecular Genetics, Faculty of Biology, Institute of Molecular Biology and Biotechnology, Adam Mickiewicz University Poznan, Uniwersytetu Poznanskiego 6, 61-614 Poznan, Poland; k.kluzek@amu.edu.pl (K.K.); h.bluyss@amu.edu.pl (H.A.R.B.); 2 Laboratory of High Throughput Technologies, Faculty of Biology, Adam Mickiewicz University Poznan, Uniwersytetu Poznanskiego 6, 61-614 Poznan, Poland; weronika.majer@amu.edu.pl (W.M.); natalia.derebecka@amu.edu.pl (N.D.); 3 Department of Urology, Poznan University of Medical Sciences, Szwajcarska 3, 61-285 Poznan, Poland; jksobkowiak@gmail.com (J.S.); mileckito@gmail.com (T.M.); zbigniew.kwias@poczta.onet.pl (Z.K.)

**Keywords:** microRNA, renal cancer, RCC, ccRCC, meta-analysis

## Abstract

**Simple Summary:**

In the majority of renal cancer cases, the disease course is non-symptomatic which frequently leads to late diagnosis of disease. Currently, there are no molecular tools dedicated to the detection and monitoring of renal cancer. Our study aimed to investigate changes in microRNA (miRNA) expression in tissue samples of renal cancer patients. We performed meta-analysis using results of 14 high-throughput studies (both, NGS and microarrays) and as a result, selected a group of miRNAs deregulated in renal cancer and its subtypes. Later, the expression changes of selected miRNA were validated in an independent sample set. We confirmed that the investigation of miRNA expression might be potentially applicable in the detection and monitoring of renal cancer and its subtypes.

**Abstract:**

Renal cell carcinoma (RCC) is one of the most common cancers worldwide with a nearly non-symptomatic course until the advanced stages of the disease. RCC can be distinguished into three subtypes: papillary (pRCC), chromophobe (chRCC) and clear cell renal cell carcinoma (ccRCC) representing up to 75% of all RCC cases. Detection and RCC monitoring tools are limited to standard imaging techniques, in combination with non-RCC specific morphological and biochemical read-outs. RCC subtype identification relays mainly on results of pathological examination of tumor slides. Molecular, clinically applicable and ideally non-invasive tools aiding RCC management are still non-existent, although molecular characterization of RCC is relatively advanced. Hence, many research efforts concentrate on the identification of molecular markers that will assist with RCC sub-classification and monitoring. Due to stability and tissue-specificity miRNAs are promising candidates for such biomarkers. Here, we performed a meta-analysis study, utilized seven NGS and seven microarray RCC studies in order to identify subtype-specific expression of miRNAs. We concentrated on potentially oncocytoma-specific miRNAs (miRNA-424-5p, miRNA-146b-5p, miRNA-183-5p, miRNA-218-5p), pRCC-specific (miRNA-127-3p, miRNA-139-5p) and ccRCC-specific miRNAs (miRNA-200c-3p, miRNA-362-5p, miRNA-363-3p and miRNA-204-5p, 21-5p, miRNA-224-5p, miRNA-155-5p, miRNA-210-3p) and validated their expression in an independent sample set. Additionally, we found ccRCC-specific miRNAs to be differentially expressed in ccRCC tumor according to Fuhrman grades and identified alterations in their isoform composition in tumor tissue. Our results revealed that changes in the expression of selected miRNA might be potentially utilized as a tool aiding ccRCC subclass discrimination and we propose a miRNA panel aiding RCC subtype distinction.

## 1. Introduction

Renal cell carcinoma (RCC) is one of ten the most commonly occurring cancer types worldwide [1]. The occurrence of RCC is population dependent, although the general incidence is estimated to be 10 per 100,000 individuals [2]. The 5-year recovery rate of metastatic RCC patients is 12.3% [3] and is frequently a consequence of a late diagnosis. Nearly non-symptomatic disease course and lack of characteristic symptoms except flank pain, hematuria and hypertension accompanied by general fatigue, recurrently lead to the identification of RCC in advanced and/or metastatic stage, with 18% of patients displaying peripheral metastases in distal organs [4]. However, first mutations leading to the tumor development occur in childhood or adolescence, years or even decades before diagnosis [5]. Since RCC is chemo- and radiotherapy resistant the main RCC treatment is partial or complete nephrectomy [6].

Four main RCC subtypes have been identified: clear cell renal cell carcinoma (ccRCC) (75–85% of all RCC cases), papillary (pRCC) (12–14%), chromophobe (chRCC) (4–6%), clear cell papillary (ccpRCC) (~4%) also accompanied by renal oncocytoma, often benign, that comprises approximately 1% of kidney tumors [6,7]. Additionally, over the last few years a relatively rare subtype of renal tumor tubulocystic RCC (tcRCC) was described [8]. The diagnosis relays mainly on the results of imaging techniques, such as computer tomography or magnetic resonance, rarely followed by a tumor biopsy. RCC subtype differentiation is confirmed after tumor resection by histological examination of the tumor slides. Frequently, RCC tumors are difficult to distinguish due to the limitations of the imaging techniques and histological classification might be incorrect due to tumor heterogeneity. In the majority of oncocytoma cases, surgical intervention is not required but incorrect tumor classification may lead to unnecessary surgery [9]. Additionally, frequent and repetitive use of imaging techniques or biopsy could be potentially harmful to patients (excess of radiation or post-procedure complications) [10]. Therefore, new methods that could aid cancer detection and RCC classification such as noninvasive molecular biomarkers are a promising alternative.

Non-invasive biomarkers have been utilized in many cancer types and include Human Epidermal Growth Factor Receptor 2 (HER2) (in breast tumors), BRAF V600E (in metastatic melanoma), Prostate Specific Antigen (PSA) (in prostate cancer) and Carcinoembryonic Antigen (CEA) (in colorectal cancer) [11,12]. On the other hand, there are no specific non-invasive biomarkers aiding RCC diagnosis. However currently, clinical trials (e.g., RECORD-3) are focused on finding non-invasive biomarkers to monitor treatment outcome [13].

In recent years, there is an increasing interest in employing microRNA (miRNA)—small noncoding approximately 22 nucleotides long RNA—as cancer biomarkers [14]. miRNA originates during a multistep process in which long miRNA transcript, called primary-miRNA (pri-miRNA) is cleaved to ~70 nt length pre-miRNA by Drosha Ribonuclease III (DROSHA) in complex with DiGeorge Syndrome Critical Region Gene 8 (DGCR8). Pre-miRNA is further cleaved by DICER1 Ribonuclease III to ~22 nt double-stranded miRNA molecule. One of these strands is loaded into Argonaut 2 protein (AGO2) creating RNA Inducing Silencing Complex (RISC), the second strand is degraded [15]. RISC takes part in posttranscriptional gene expression by blocking mRNA translation or initiating mRNA cleavage [16] due to the presence of “seed” sequence in miRNA, complementary to mRNA usually in 3′ untranslated region (3′UTR) of targeted mRNA. Mature miRNA can occur in isoforms (iso-miRNA) processed from the same pri-miRNA and different at 5’ and 3’ ends as a result of inaccurate cleavage by DROSHA and DICER1. Those modifications can influence miRNA activity and function. Additionally, 3’ end of miRNA may be adenylated or uridylated which affects its stability. Deregulations of miRNA expression have been previously correlated with changes in protein levels engaged in proliferation, motility or cell invasiveness and in consequence promotion of tumor development and growth [17,18].

Changes in miRNA expression are well known in RCC tumors and these abnormalities can be potentially useful to distinguish RCC subtypes, although certain discrepancies between the studies can be noted [19]. The most commonly identified as downregulated in ccRCC tumor samples were miR-141, miRNA-200c [19]. On the other hand, many studies identified miRNA-210, miRNA-224 and miRNA-155 as upregulated in ccRCC tumors [20,21]. It has been also shown ccRCC and pRCC display significant changes in miRNA-424 expression, which could be helpful in RCC tumor subtype classification [22].

Here, we performed a meta-analysis of miRNA expression in ccRCC, pRCC and chRCC tumors, analyzed the expression of miRNA isoforms and examined potential causes of miRNA deregulation in ccRCC using a bioinformatics approach. After validation of miRNA expression in RCC tumors kidney tissue, we postulate that a miRNA panel could be potentially a powerful RCC classification tool and miRNA profile may be indicative of disease grades.

## 2. Materials and Methods

### 2.1. Sample Preparation

One hundred and fifteen samples of cancer tissue and 36 adjacent noncancerous kidney samples were obtained from renal cancer patients after partial or complete nephrectomy. Samples were collected the Department of Urology and Urological Oncology, Poznan University of Medical Sciences, Poland with the signed consent of patients (bioethical consent of Local Bioethical Committee at Poznan University of Medical Sciences, no. 1124/12). Tumors were classified as RCC subtypes and according to Fuhrman grade. ccRCC tumors (*n* = 97) were divided into Fuhrman grade 1, *n* = 12; grade 2, *n* = 35; grade 3, *n* = 33; grade 4, *n* = 17. 10 pRCC, 10 oncocytoma and 36 adjacent, histopathologically unchanged kidney tissue samples were also included in the analysis (Table S1). Samples were randomly divided into groups used in different experiments (details in Table S1). The average age of patients was 65. For the preservation of RNA, tissue fragments were collected into tubes containing RNAlater (Sigma Aldrich, St. Louis, MO, USA) and stored at −80 °C for further processing. Next, fragments of tissue were transferred into a sterile mortar and grinded in liquid nitrogen. RNA was extracted with Trizol and quantified using NanoDrop ND-1000.

### 2.2. Library Preparation and Sequencing

For library preparation, 1 µg of total RNA with RNA Integrity Number (RIN) equal to or above 7 was used. RNA-Seq libraries (controls, *n* = 17; ccRCC, *n* = 58 samples) were prepared with TruSeq RNA Library Preparation Kit v2 (Illumina, San Diego, CA, USA) and small RNA-Seq libraries (controls, *n* = 6; ccRCC, *n* = 26) with TruSeq Small RNA Library Preparation Kit (Illumina, San Diego, CA, USA). Quality and concentration of the libraries were tested using Agilent DNA 1000 Kit and Agilent High Sensitivity DNA Kit (Agilent Technologies Inc., Santa Clara, CA, USA). The libraries were sequenced on HiScan SQ (Illumina) with TruSeq PE Cluster Kit v3-cBot-HS (Illumina, San Diego, CA, USA, cat. no. PE-401-3001) in PE100 and SR50 modes, respectively.

The data discussed in this publication have been deposited in NCBI’s Gene Expression Omnibus [23] and are accessible through GEO Series accession number GSE151428 (https://www.ncbi.nlm.nih.gov/geo/query/acc.cgi?acc=GSE151428).

### 2.3. Small RNA-Seq Data Processing

In small RNA-Seq analysis workflow, raw reads were trimmed with cutadapt (https://cutadapt.readthedocs.io/en/stable/), untrimmed reads and reads shorter than 10 nt were discarded. Reads were aligned to Ensembl GRCh38 human genome with bowtie2 [24]. Raw read counts were generated with featureCounts v1.6.3 [25] and differentially expressed miRNA in ccRCC tumors were identified with edgeR (v3.28) package (R v3.6). miRNA isoforms were calculated with MIRALIGNER protocol [26] and isomiRs (v1.14) (https://bioconductor.org/packages/isomiRs/) package (R v3.6) based on miRBase v22.1.

### 2.4. RNA-Seq Data Processing

RNA-Seq raw paired-end reads were trimmed with cutadapt. Trimmed reads were aligned to Ensembl GRCh38 human genome with STAR (v2.7) [27] and counts were obtained using featureCounts v1.6.3 [25]. Differentially expressed mRNA in ccRCC tumors were identified with edgeR (v3.28) package (R v3.6).

### 2.5. Meta-Analysis of miRNA Expression in RCC Tumors

Sequencing data from Exp1 were generated in our laboratory. Exp2 [28], Exp3 [29] and Exp4 [30] were downloaded from the SRA database [31] as raw reads. All small RNA-Seq experiments used in the meta-analysis were performed on fresh tissues, available Formalin-Fixed Paraffin-Embedded experiments were excluded. Exp5–Exp14 results were derived from dbDEMC2.0 database [32] (Figure S1). Significantly deregulated miRNAs in ccRCC obtained from all above experiments were compared using the dplyr package (v0.8.3; R v3.6). Venn graphs were created with the VennDiagram package (v1.6; R v3.6).

### 2.6. Poly(A)-RT

Synthesis of cDNA by polyadenylation reverse transcription reaction (Poly(A)-RT) was described previously [33]. 12.4 µL of RNA sample (1 µg of RNA) were added to reverse transcription mix containing: 12 µL of RT buffer (25 µL 1 M Tris-HCl, pH = 8.0; 93.75 µL 2 M KCl; 250 µL 100 mM DTT; 175 µL 1 M MgCl_2_); 20 µL 100 µM anchor RT primer, containing universal adapter sequence; 436.25 µL H_2_O; 6 µL deoxynucleotide mix (100 mM of each); 25 µL 10 mM rATP; 25 µL 40 U/µL RiboLock (ThermoFisher, Waltham, MA, USA); 0.6 µL *E. coli* poly(A) polymerase (New England Biolabs, Rowley, MA, USA) and 0.6 µL reverse transcriptase (ThermoFisher). The reaction was performed at 37 °C for 1 h followed by inactivation at 85 °C for 10 min.

### 2.7. qPCR

In the analysis of mRNA and miRNA expression, 2 μL of the 5 times diluted cDNA template were used per qPCR reaction. miRNA amplification was performed with miRNA specific forward primer (Table S4) and universal reverse primer complement to the adapter sequence. In all reactions Maxima SYBR Green/ROX qPCR Master Mix (2X) (ThermoFisher) was used. miRNA results were normalized to U6. Specific “iso-miRNA primers,” which discriminate miRNA-363-3p or miRNA-224-5p shorter isoforms and amplify longer isoforms, were used in qPCR. Iso-miRNA results were normalized to U6 or specific miRNA. mRNA results were normalized to *GAPDH*. The expression level was determined by the 2^−∆∆Ct^ method [34].

### 2.8. Gene Ontology (GO) Analysis of miRNA Targets

Gene Ontology analysis for miRNA targets was performed with GeneMANIA (v3.5.1) [35] in Cytoscape (v3.7.1) [36]. The most probable miRNA targets clusters in networks were detected with MCODE (v1.5.1) [37] in Cytoscape (v3.7.1).

### 2.9. Statistical Analysis

Pearson correlation coefficient was calculated and the regression graph was created with base functions of R (v3.6). The remaining statistical analyses were performed using a two-tailed *t*-test in Microsoft Excel (NS, non-significant; * *p* < 0.05; ** *p* < 0.01 and *** *p* < 0.001), ± standard error of the mean (SEM). The number of biological replicates (n) is shown in the figure descriptions. Receiver operating characteristic (ROC) analysis was performed with the MetaboAnalyst tool [38]. Kaplan-Meier survival plots were created based on The Cancer Genome Atlas (TCGA) data with Kaplan-Meier Plotter [39].

## 3. Results

### 3.1. Small RNA-Seq and Meta-Analysis

In order to identify specifically deregulated microRNAs in RCC first we conducted small RNA-Seq experiment on ccRCC tumor tissue derived from Polish patients (Exp1, ccRCC: *n* = 26, controls: *n* = 6). The data was extended with additional publicly available data sets, derived from both NGS and microarray experiments, collected from Sequence Read Archive (SRA) and dbDMEMC 2.0 databases. The details of the experiments and analytic workflow were listed in Figure S1.

The NGS data was collected in form of raw reads, subjected to the identical data processing and included four small RNA-Seq experiments performed on ccRCC tumors (Exp1–Exp4) [28,29,30]. The final number of differentially expressed miRNAs (FDR < 0.05) differed per data set (Figure S1a).

Additionally, in order to retrieve the information derived from NGS experiments collected by The Cancer Genome Atlas (pRCC, chRCC) and microarray experiments (pRCC, chRCC and oncocytoma), we utilized datasets from the dbDEMC 2.0 in a form of lists of differentially expressed (DE) miRNAs. The data included: four ccRCC (Exp5–Exp8) [40,41,42], three pRCC (Exp9–Exp11) [40,43], two chRCC (Exp12, Exp13) [40] and one oncocytoma (Exp14) [40] experiments (Figure S1a).

**Figure 1:**
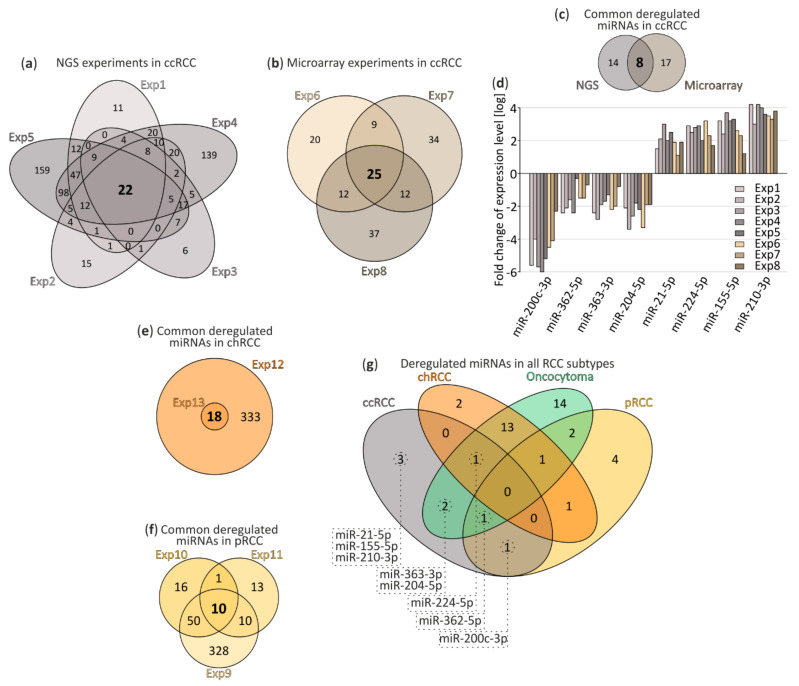
The meta-analysis of deregulated miRNA in renal cell carcinoma (RCC) tumor tissues. (**a**,**b**) Venn diagrams depicting commonly deregulated miRNAs in clear cell renal cell carcinoma (ccRCC) tumors. (**a**) small RNA-seq and (**b**) microarray experiments; (**c**,**d**) Comparison of commonly deregulated miRNA in chromophobe renal cell carcinoma (chRCC) (**c**) and papillary (pRCC) (**d**); (**e**) miRNA identified in both next-generation sequencing (NGS) and microarray experiments performed in ccRCC; (**f**) Comparison of eight miRNA expression levels commonly disrupted in ccRCC reported in original NGS and microarray experiments; (**g**) Comparison of commonly deregulated miRNA in ccRCC (8 miRNA), chRCC (18 miRNA), oncocytoma (34 miRNA) and pRCC (10 miRNA). ccRCC, clear renal cell carcinoma; chRCC, chromophobe renal cell carcinoma; pRCC, papillary renal cell carcinoma; Exp1–8, experiments 1–8.

After data processing, we performed a meta-analysis of deregulated miRNAs in ccRCC and compared significantly deregulated miRNAs in NGS experiments (Figure 1a) and microarrays (Figure 1b) identified 22 and 25 commonly deregulated miRNAs, respectively. Due to technical and analytical differences between compared experiments, we implemented stringent exclusion criteria: only miRNAs reported in all data sets to be deregulated, with significant FDR values were taken into account. chRCC was characterized by deregulation of 18 miRNAs (Figure 1e) and 10 miRNAs were found deregulated in pRCC (Figure 1f). Limited information on oncocytoma listed 34 deregulated miRNAs (single microarray experiment). Detailed lists of DE miRNAs, including FC and FDR parameters, are included in Supplementary File S1.

After comparison of all available data sets eight commonly deregulated miRNAs in ccRCC were selected (Figure 1c) and those included: miR-200c-3p, miR-362-5p, miR-363-3p and miR-204-5p as downregulated and miR-21-5p, miR-224-5p, miR-155-5p and miR-210-3p as upregulated (Figure 1d).

Interestingly, as depicted in Figure 1g, our analysis suggests that there are no commonly deregulated miRNAs for all RCC tumors, although we cannot exclude the possibility of missing a number of miRNA candidates due to rigorous cut-off criteria. Additionally, this analysis suggests that deregulation of miR-21-5p, miR-155-5p and miR-210-3p could be ccRCC-specific.

### 3.2. Validation of RCC-Specific miRNA Candidates

Next, we set out to validate the expression of potentially RCC subtype-specific miRNAs in an independent sample set using quantitative PCR (qPCR). Due to lack of availability of chRCC samples the 18 commonly deregulated miRNAs were not verified. We included 39 ccRCC, 10 pRCC and 8 oncocytoma tumor tissues, with 15 adjacent, histopathologically unchanged kidney tissue samples.

From potentially pRCC- or oncocytoma-specific miRNAs distinguished in meta-analysis (Figure 1 and Supplementary File S1) those with the highest fold change were selected to further validation.

**Figure 2:**
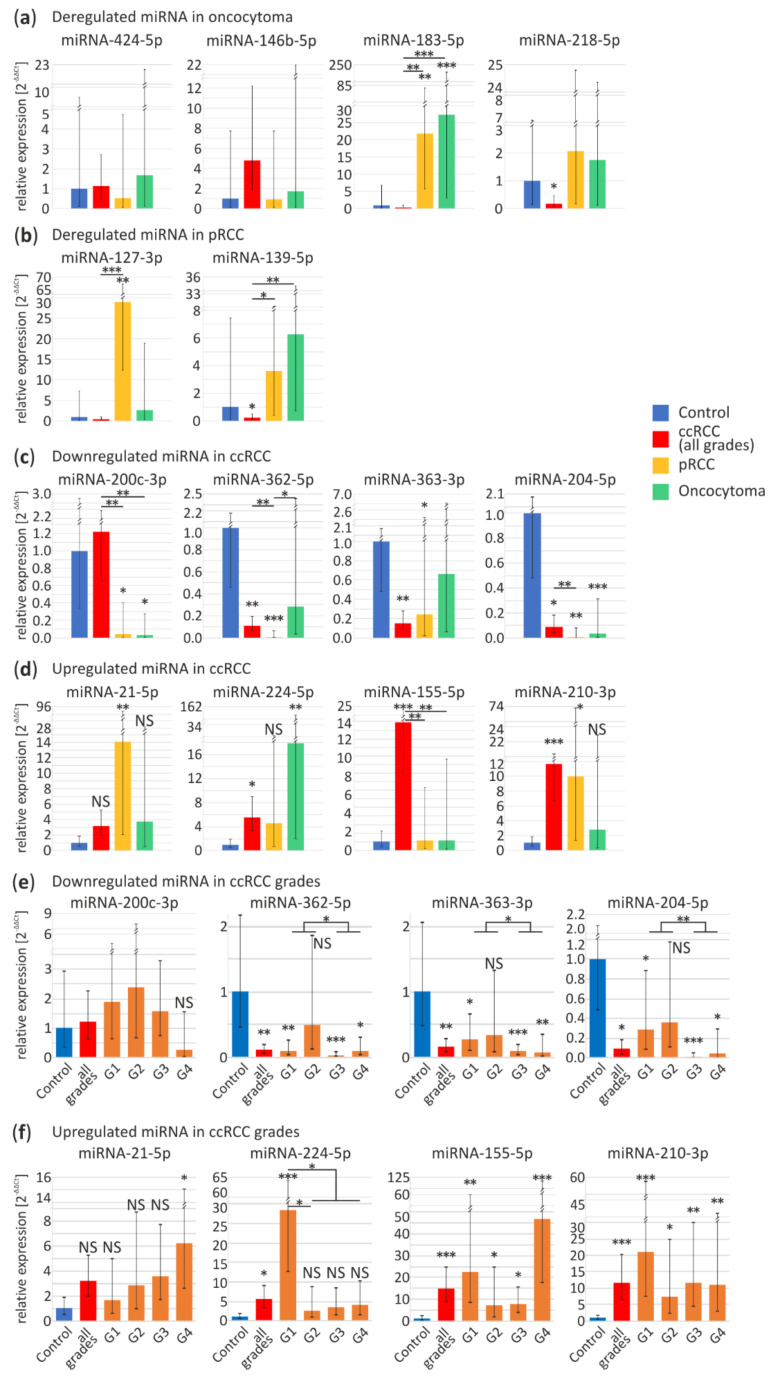
Validation of commonly deregulated miRNA in the independent sample set of ccRCC, pRCC and oncocytoma tumors by quantitative PCR (qPCR). (**a**,**b**) Specifically deregulated miRNA in oncocytoma (**a**) and pRCC (**b**) tumors based on a meta-analysis compared to other subtypes. Analyzed specimens included: control (*n* = 9), ccRCC (*n* = 11), pRCC (*n* = 10), oncocytoma (*n* = 8) samples; (**c**–**f**) Validation of expression disruptions of commonly deregulated miRNA in RCC tumors. Downregulated (**c**) and upregulated (**d**) miRNA in ccRCC tumors based on a meta-analysis compared to pRCC and oncocytoma. Analyzed specimens included: control (*n* = 15), ccRCC (*n* = 39), pRCC (*n* = 11), oncocytoma (*n* = 8) samples; (**e**,**f**) miRNA expression in ccRCC tumors grouped according to Fuhrman grade: downregulated (**e**) and upregulated (**f**) miRNA in ccRCC tumors based on a meta-analysis. Analyzed specimens included: control, *n* = 15; ccRCC (*n* = 39), ccRCC G1 (*n* = 9), ccRCC G2 (*n* = 11), ccRCC G3 (*n* = 10), ccRCC G4 (*n* = 9) samples. Blue bars, control tissue; red bars, ccRCC tumors; orange bars, ccRCC Fuhrman grades; yellow bars, pRCC; green bars, oncocytoma; ccRCC, clear renal cell carcinoma; pRCC, papillary renal cell carcinoma; G1-G4, ccRCC Fuhrman grades 1–4. NS, non-significant; * *p* < 0.05; ** *p* < 0.01 and *** *p* < 0.001.

For oncocytoma-specific miRNA validation we selected four miRNAs: downregulated miRNA-424-5p, miRNA-146b-5p and upregulated miRNA-183-5p and miRNA-218-5p. In independent sample-set miRNA-183-5p is upregulated in pRCC (22-fold change, *p* < 0.01) and oncocytoma (27-fold change, *p* < 0.001) samples, while level of miRNA-218-5p is significantly decreased in ccRCC tumors (0.17-fold change, *p* = 0.048) (Figure 2a). miRNA-424-5p, miRNA-146b-5p display similar expression levels in all samples. Our findings suggest that miRNAs selected in the meta-analysis are not oncocytoma-specific.

According to high-throughput data miRNA-127-3p and miRNA-139-5p should be downregulated in pRCC tumors. Although, in contrast to previous reports, in our sample set miRNA-127-3p appears to be significantly upregulated in pRCC (29-fold change, *p* < 0.01). Increase in expression of miRNA-139-5p does not significantly differ in pRCC and oncocytoma (Figure 2b), however miRNA-139-5p appears to be downregulated in ccRCC (0.2-fold change, *p* = 0.04). Inconsistency of these data suggest rejection of these miRNA as pRCC-specific.

As shown in Figure 2c,d, expression profiles of eight potentially ccRCC-specific microRNAs were further investigated. Remarkably, downregulation of miR-200c-3p is observed in pRCC (0.04-fold change, *p* < 0.014) and oncocytoma (0.03-fold change, *p* = 0.03) with no change in ccRCC (for explanation see below). The remaining miRNAs do not appear to be ccRCC-specific. Although, read out of the miR-362-5p (ccRCC, 0.1-fold change, *p* < 0.01; pRCC, 0.007-fold change, *p* < 0.001) and miR-363-3p (ccRCC, 0.15-fold change, *p* < 0.01; pRCC, 0.2-fold change, *p* = 0.03) did not reach the statistical significance in oncocytoma. Only miR-204-5p, was significantly downregulated in all RCC tumor types as compared to controls (ccRCC, 0.09-fold change, *p* = 0.011; pRCC, 0.006-fold change, *p* < 0.01; oncocytoma, 0.03-fold change, *p* < 0.001).

miRNA-21-5p was overexpressed solely in pRCC tumors (14-fold change, *p* < 0.01). Levels of both miRNA-224-5p and miRNA-210-3p were found significantly increased in ccRCC tumors (5.5-fold change, *p* = 0.02; 12-fold change, *p* < 0.001, respectively), although they were also upregulated in oncocytoma (18-fold change, *p* < 0.01) and pRCC (10-fold change, *p* = 0.013), respectively. There was no significant change in the expression of miR-21-5p, miR-224-5p and miR-210-3p among the subtypes (Figure 2d). Interestingly, significant miR-155-5p upregulation appeared to be distinctive of ccRCC (15-fold change, *p* < 0.001) (Figure 2d).

Next, we analyzed expression profiles of the eight miRNAs in ccRCC tumors with different grading, represented by nine tumors in Fuhrman grade one (G1), eleven in grade two (G2), ten in grade three (G3) and nine in grade four (G4). Figure 2e shows no significant changes in different tumor grades in case of miRNA-200c-3p. miRNA-200c-3p is one of most commonly identified as ccRCC downregulated miRNA [44]. Since we could not validate its downregulation, although also suggested by NGS data implemented in the meta-analysis, we investigated the potential reasons for such discrepancy. Firstly, we compare sequences of miR-200 family members including miR-200a, miR-200b, miR-200c, miR-141 and miR-429 (Figure S2a). Due to a significant sequence homology between the miRNA-200 family members, we hypothesized that simultaneous amplification of more than one miRNA might be the reason for the inconsistency between the studies. To further support this hypothesis, we investigated the contribution of miR-200c-3p in the complete family which is relatively low in both control and ccRCC tissues based on NGS data (Figure S2b). Additionally, the comparison of miR-200 family members expression in all available data sets shows that they could be deregulated in all RCC tumor types (Figure S2c). These observations confirming the potential cumulative readout might lead to misinterpretation of the data provided by qPCR.

Consistent downregulation throughout all tumor grades were observed in case of miR-362-5p (G1, 0.09-fold change, *p* < 0.01; G3, 0.03-fold change, *p* < 0.001; G4, 0.09-fold change, *p* = 0.013), miR-363-3p (G1, 0.3-fold change, *p* = 0.019; G3, 0.08-fold change, *p* < 0.001; G4, 0.07-fold change, *p* < 0.01) and miR-204-5p (G1, 0.3-fold change, *p* = 0.042; G3, 0.01-fold change, *p* < 0.001; G4, 0.05-fold change, *p* = 0.02) (Figure 2e). Significant upregulation of miRNA-21-5p expression is present only in the G4 (6-fold change, *p* = 0.047), although, increasing expression of this miRNA is noticeable across all Fuhrman grades (Figure 2f). The following miRNAs displayed elevated expression in all samples: miR-210-3p (G1, 21-fold change, *p* < 0.001; G2, 7.4-fold change, *p* = 0.013; G3, 12-fold change, *p* < 0.01; G4, 11-fold change, *p* < 0.01) and miR-155-5p (G1, 22-fold change, *p* < 0.01; G2, 7.4-fold change, *p* = 0.02; G3, 8-fold change, *p* = 0.03; G4, 47-fold change, *p* < 0.001). Interestingly, significant rise of miR-155-5p was clearly observed in G4 (Figure 2f). Interestingly, significant miR-224-5p overexpression was characteristic only for G1 ccRCC tumors (28-fold change, *p* < 0.001).

Validation of expression levels of eight selected miRNA revealed grade-dependent variations in ccRCC tumor tissues, whereas, a comparison of expression levels of the 14 miRNAs between ccRCC, pRCC and oncocytoma suggests differences in miRNA expression dependent on the RCC subtype.

### 3.3. ROC Analysis

**Figure 3:**
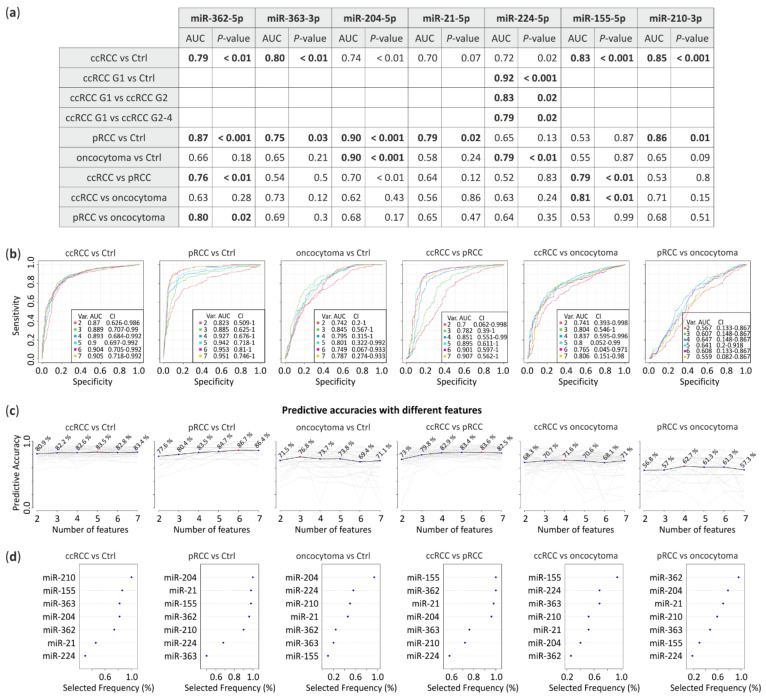
Receiver Operating Characteristic (ROC) analysis predictive potential of selected miRNA. (**a**) Area under the curve (AUC) with *p*-value calculated from miRNA expression level obtained from qPCR. Bold, comparisons with AUC > 0.75 and *p*-value < 0.05; (**b**) ROC multivariate analysis with seven proposed miRNAs; (**c**) Predictive accuracies with different miRNAs (red dot indicates a number of features with the highest predictive accuracy) and (**d**) frequency of selection in the test.

Receiver operating characteristic (ROC) analysis was performed to assess the prognostic accuracy of the miRNA signatures based on real-time PCR results. The area under the curve (AUC) was calculated for each comparison. miRNA expression levels obtained from all RCC subtypes were compared with control tissue or between each other to calculate their predictive potential (Figure 3). The data suggest that downregulated miRNA-362-5p (AUC = 0.79, *p* < 0.01) and miRNA-363-3p (AUC = 0.8, *p* < 0.01) jointly with upregulated miRNA-155-5p (AUC = 0.83, *p* < 0.001) and miRNA-210-3p (AUC = 0.85, *p* < 0.001) significantly differentiates ccRCC tumors from healthy tissue. pRCC could be classified using miRNA-362-5p (AUC = 0.87, *p* < 0.001), miRNA-363-3p (AUC = 0.75, *p* = 0.03), miRNA-204-5p (AUC = 0.9, *p* < 0.001), miRNA-21-5p (AUC = 0.79, *p* = 0.02) and miRNA-210-3p (AUC = 0.86, *p* = 0.01) while oncocytoma with miRNA-204-5p (AUC = 0.9, *p* < 0.001) and miRNA-224-5p (AUC = 0.79, *p* < 0.01). Furthermore, ccRCC could be significantly distinguished from pRCC with miRNA-362-5p (AUC = 0.76, *p* < 0.01) and miRNA-155-5p (AUC = 0.79, *p* < 0.01) or from oncocytoma with miRNA-155-5p (AUC = 0.81, *p* < 0.01). Whereas, miRNA-362-5p (AUC = 0.8, *p* = 0.02) differentiates pRCC and oncocytoma. Additionally, expression changes of miRNA-224-5p shows potential to discriminate ccRCC Fuhrman grades, G1 versus G2 with AUC = 0.83 (*p* = 0.02) or G1 versus G2-4 with AUC = 0.79 (*p* = 0.02) (Figure 3a).

To propose potential panels of miRNA to distinguish RCC subtypes we performed ROC multivariate analysis (Figure 3b). Predictive accuracies with different miRNAs (Figure 3c) suggest that 5, 6 and 3 miRNAs distinguish ccRCC, pRCC and oncocytoma from healthy tissue with 83.5%, 86.7% and 76.8% of accuracy, respectively. RCC subtypes could be differentiated by 6 miRNAs (ccRCC vs. pRCC, 83.6% of accuracy), 4 miRNAs (ccRCC vs. oncocytoma, 71.6% of accuracy) or 4 miRNAs (pRCC vs. oncocytoma, 62.7% of accuracy). Selection frequency of each miRNA in the ROC test is shown on Figure 3d.

Our data suggest that proposed miRNA could have diagnostic potential and could efficiently distinguish RCC subtypes or between ccRCC tumors with Fuhrman grades.

### 3.4. Iso-miRNA Analysis

Mature miRNA could occur in isoforms that vary in length or presence of poly(A) or poly(U) tails on 3’ end. According to literature, iso-miRNA expression levels could successfully differentiate cancer types [45]. Hence, we decided to investigate iso-miRNA signatures in ccRCC using data sets obtained from Exp1 and Exp4 and validate them in ccRCC (controls, *n* = 4; ccRCC, *n* = 17).

**Figure 4:**
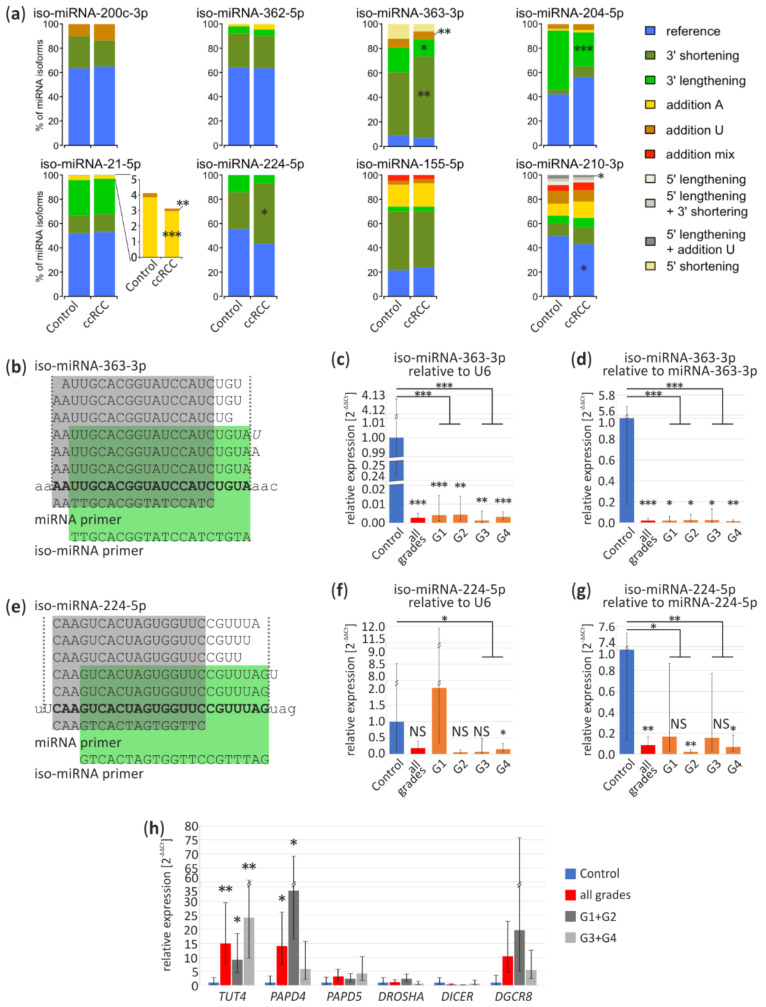
miRNA isoform deregulation in ccRCC tumors. (**a**) The percentage per miRNA isoform (iso-miRNA) in control and tumor samples based on Exp1 results. Part of the chart represents the percentage of iso-miRNA-21-5p is magnified to show more accurately 3′ tailing of miRNA-21-5p; (**b**,**e**) Sequences of miRNA-363-3p and miRNA-224-5p isoforms identified in kidney, respectively. “miRNA primer” (in grey) amplifies all iso-miRNA and “iso-miRNA primer” (in green) amplifies reference miRNA and longer iso-miRNA. Bold, reference miRNA sequence; small letters, pre-miRNA sequence; italic, additional U or A nucleotides; (**c**,**d**) Validation of iso-miRNA-363-3p in ccRCC tumors and controls by qPCR relative to U6 (**c**) and miRNA-363-3p (**d**); (**f**,**g**) Validation of iso-miRNA-224-5p in ccRCC tumors and controls by qPCR relative to U6 (**f**) and miRNA-224-5p (**g**); (**h**) qPCR analysis of expression of genes involved in miRNA processing and after maturation modifications in ccRCC tumors. Control (*n* = 4), ccRCC (*n* = 17) containing: G1 (*n* = 4), G2 (*n* = 3), G3 (*n* = 5), G4 (*n* = 5). NS, non-significant; * *p* < 0.05; ** *p* < 0.01 and *** *p* < 0.001.

We analyzed the percentage contribution of iso-miRNA of eight miRNAs, which according to our meta-analysis were commonly deregulated in ccRCC (Table S2). As shown in Figure 4a (upper panel), among four downregulated miRNAs, two: miR-363-3p and miR-204-5p exhibit significantly different isoform expression pattern in ccRCC tumors. In case of miR-363-3p, shortening of 3′ end is more frequent with simultaneous reduction of elongation, with 67% and 14% contribution as compared to control: 52% and 20%, respectively (*p* < 0.01 and *p* = 0.047, respectively). Similarly, poly(U) addition is more common in non-ccRCC tissue (control 15%, ccRCC 7.3%; *p* < 0.01). We also observed more reduced 3′ end lengthening of miRNA-204-5p in ccRCC (control 49%, ccRCC 28.5%; *p* < 0.001).

In the group of potentially ccRCC-specific, upregulated miRNAs (Figure 4a lower panel) miRNAs miR-21-5p displayed slight, less frequent, though statistically significant modifications, with addition of poly(A) (control 4%, ccRCC 3%; *p* < 0.001) and poly(U) (control 0.3%, ccRCC 0.2%). In case of miR-224-5p 3′ lengthening has elevated level in ccRCC (control 30%, ccRCC 50%; *p* < 0.01). Reference miRNA-210-3p has significantly lower percentage by 7% in ccRCC (*p* = 0.01). Interestingly, in case of miRNA-210-3p, we found differences in sequencing Exp1 and Exp4 concerning a tendency toward 5’ lengthening and less frequent addition of U nucleotide (Exp1, *p* = 0.02) and more frequent addition of mixed nucleotides in Exp4 (*p* = 0.049), which inclines us to carefully interpret the sequencing data and stresses the necessity for in depth, a multi-dataset study on miRNA isoforms (Figure 4a and Figure S3a).

Specific primers used in qPCR (“iso-miRNA primer”) (Figure 4b,e) discriminate miRNA-363-3p and miRNA-224-5p shorter isoforms and amplify longer isoforms. As shown on an independent sample set (Figure 4c), iso-miRNA-363-3p expression was relatively similar in all tumors, regardless their grading status (G1, 0.004-fold change, *p* < 0.001; G2, 0.004-fold change, *p* < 0.01; G3, 0.001-fold change, *p* < 0.01; G4, 0.003-fold change, *p* < 0.001). Interestingly, iso-miRNA-224-5p was the most significant downregulated in G4 (0.15-fold change, *p* = 0.02) (Figure 4f), in contrast to miRNA-224-5p upregulation in G1 (Figure 3d). In relation to miR-363-3p (G1, 0.02-fold change, *p* = 0.01; G2, 0.02-fold change, *p* = 0.01; G3, 0.02-fold change, *p* = 0.01; G4, 0.01-fold change, *p* < 0.01) and miR-224-5p (G2, 0.02-fold change, *p* < 0.01; G4, 0.07-fold change, *p* = 0.02) both iso-miRNAs are significantly reduced in ccRCC (Figure 4d,g) which confirms decreased level of longer isoforms (Figure 4a).

Additionally, we analyzed expression profiles of genes involved in miRNA maturation (*DROSHA*, *DGCR8*, *DICER1*) or post-maturation processing (Terminal Uridylyl Transferase 4, *TUT4*; PAP Associated Domain Containing 4, *PAPD4;* PAP Associated Domain Containing 5, *PAPD5*) (Figure 4h). qPCR results suggest that *TUT4* and *PAPD4* are significantly upregulated in ccRCC tumors (15-fold change, *p* < 0.01 and 14-fold change, *p* < 0.05, respectively), however, expression of *PAPD4* is the highest in tumors with lower grades (G1 + G2) (34-fold change, *p* = 0.03).

Our data suggest that iso-miRNA contribution in ccRCC tumors may differ from the control tissue and observed expression shifts if validated, could aid ccRCC classification. Additionally, disruptions of miRNA expression in ccRCC could be partially explained by differences in miRNA isoform stability.

### 3.5. Basis of Deregulation of Selected miRNA in ccRCC

In order to investigate the character of deregulation of the eight commonly disrupted miRNAs in ccRCC we explored the possibility of co-transcriptional deregulation of miRNAs with their host genes. We utilized the data from RNA-Seq, performed on ccRCC tumors (ccRCC: *n* = 60, controls: *n* = 17) matching small RNA-Seq samples (this work). As shown in Figure S3b miR-200c, miR-204 and miR-362 host genes (*MIR200CHG*, *TRPM3*, *CLCN5*, respectively) display a statistically significant decrease in their expression. Similarly, miR-224, miR-21, miR-155 and miR-210 host genes (*GABRE*, *VMP1*, *MIR155HG* and *MIR210HG*, respectively), are elevated, supporting the mechanism of co-transcriptional regulation being the basis of seven out of eight analyzed miRNAs. The expression of miRNAs and their host genes was highly correlated, with *R* = 0.97 and statistically significant (*p* < 0.001) (Figure S3c). Since miR-363 is encoded by an intergenic locus, no data was obtained from RNA-Seq.

These data suggest that miRNAs are deregulated co-transcriptionally in ccRCC tumors. Although, more studies are necessary to investigate factors responsible for this disruption.

### 3.6. miRNA Functions

**Figure 5:**
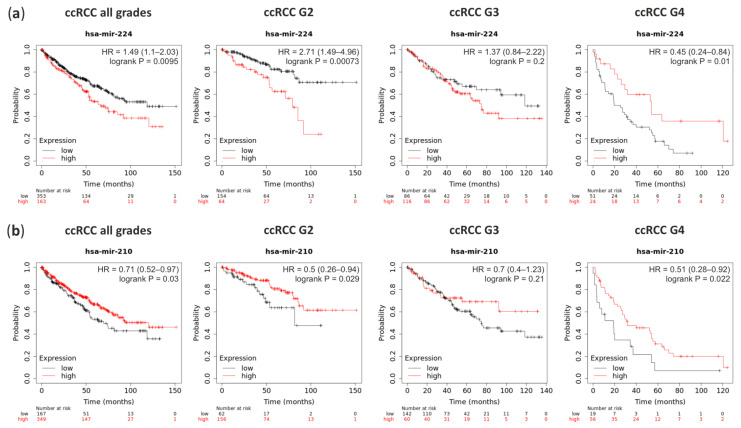
The survival rate analysis of ccRCC patients with deregulation expression of miRNA-224 and miRNA-210. (**a**) Patients with high expression (red line) of miRNA-224 have worst hazard ratio (HR) in ccRCC G2 than in G4; (**b**) Patients with high expression (red line) of miRNA-210 have higher survival rate although its massive upregulation in ccRCC tumors.

Survival analysis performed on the TCGA data with Kaplan-Meier Plotter on-line tool revealed that ccRCC patients with a high level of miRNA-224 (which is overexpressed in ccRCC G1) significantly classified patients into higher risk for death (hazard ratio (HR) = 1.49) (Figure 5a). Although, higher grades patients show the opposite pattern. In contrary, patients with the high level of miRNA-210, which is upregulated in ccRCC, have a lower risk for death (HR = 0.71) which is independent of the ccRCC Fuhrman’s grade (Figure 5b).

One miRNA could regulate the expression of many genes and simultaneously one mRNA could be targeted by a few miRNAs. Using data from the miRTarBase database [46], we obtained a list of validated gene targets of 8 commonly deregulated miRNA in ccRCC. Subsequently, we selected genes expressed in the kidney, examined their expression using RNA-seq data from tumors derived from Polish ccRCC patients (Supplementary File S2) and followed with gene ontology enrichment analysis (GO) using GeneMANIA application in Cytoscape.

The majority of identified pathways for example, cellular response to hypoxia, response to TGF-beta, serine/threonine kinase signaling pathway are well known to be de-regulated in ccRCC, although our analysis points to other, also interesting pathways that may contribute to ccRCC etiology, involving components of the homotypic fusion and protein sorting (HOPS)-tethering complex and mRNA poly(A) tail shortening (Table 1 and Table S3).

From networks created with GeneMANIA, we extracted the most crucial clusters (the most connected regions) of genes and networks by MCODE application. On these networks, we overlaid fold change and significance of differently expressed genes in ccRCC tumors based on RNA-Seq results as shown in Figure S4. These data suggest that targets of the selected miRNAs are involved in crucial pathways for ccRCC development such as cellular response to hypoxia, chromosome segregation or response to signaling factors (Table 1).

## 4. Discussion

Taking into consideration the asymptomatic course of RCC, its frequent diagnosis at advance stage and as consequence its relatively low 5-years survival rate, effective clinical and molecular markers aiding RCC classification, detection and monitoring could significantly improve disease management. Molecular alterations in RCC tumors have been extensively studied in the last decade, especially in ccRCC but neither identified mutations (e.g.,: *VHL*, *BAP1, PBRM1*, *SETD2*, *KDM5C*, *MTOR*) nor transcriptome-based ccRCC tumor sub-classification have straightforward clinical relevance [47,48]. Chromosomal rearrangements which are ccRCC drivers occur decades before diagnosis in childhood or adolescence which makes early detection of the diseases difficult [5]. The tools suitable for detection of disease initiation, early diagnosis and progression are currently not available, hence the need for identification of novel, molecular and ideally, noninvasive biomarkers.

As miRNA are short molecules, relatively stable in tissues and body fluids and have been previously shown to be deregulated in all RCC subtypes. miRNA panel could be utilized as a subtype classification, an indicator of a disease stage or treatment monitoring tool. Furthermore, miRNA regulate the expression of thousands of genes, therefore their deregulation likely plays a role in ccRCC pathogenesis.

We set off to perform a meta-analysis of miRNA expression in ccRCC, chRCC, pRCC and oncocytoma, using publicly available data sets derived from small RNA-Seq and microarray experiments. As a result, we obtained a list of miRNAs commonly deregulated in ccRCC, chRCC and pRCC. In the case of oncocytoma, only one study was available (Figure 1). Based on the meta-analysis ccRCC could be potentially classified by the comparison of expression levels of eight miRNAs: miRNA-200c-3p, miRNA-362-5p, miRNA-363-3p, miRNA-204-5p, miRNA-21-5p, miRNA-224-5p, miRNA-155-5p and miRNA-210-3p. miRNAs identified as specifically expressed in ccRCC, pRCC and oncocytoma subtypes were validated in an independent sample set (Figure 2).

All eight commonly deregulated miRNAs in ccRCC were previously described in the literature as onco-suppressors or oncogenes in various cancer types [49,50,51]. Several studies showed that miRNA-21-5p is upregulated in solid cancers, mainly in advanced tumors and has been linked to uncontrolled cell growth and necrosis [52]. Recently miRNA-21-5p was suggested as a potential therapeutic target, likely involved in processes of drug resistance in breast cancer and leukemia [53]. miRNA-224-5p was reported to be upregulated for example in colorectal cancer and hepatocellular carcinoma. However, its downregulation also was observed in prostate cancer [54,55]. This miRNA was shown to regulate cell signaling, proliferation and response to fibroblast and epidermal growth factors. miRNA-155-5p, which is upregulated in the majority of solid tumors [56,57], target genes are involved in tumorigenesis, DNA damage repair and inflammation. Elevated expression of miRNA-155-5p induces the formation of new blood vessels and tumor growth [58,59]. Furthermore, miR-155-5p influences hypoxia by targeting *VHL* mRNA [59] and its overexpression is additionally connected to diminished drug response and chemo- and radio-resistance of breast and colon cancer cells [57,59]. Overexpression of miRNA-210-3p correlates with a negative disease outcome in several cancers [15]. Many miRNA-210-3p targets are engaged in angiogenesis, cell survival and differentiation [15] miRNA-200c-3p is one of the most significantly downregulated miRNAs in ccRCC tumors [44]. miRNA-200c is member of miRNA-200 family (miRNA-200a, miRNA-200b, miRNA-200c, miRNA-141 and miRNA-429), commonly deregulated in other cancer types. miRNA-200c-3p targets are engaged in cell signaling, proliferation, cell invasion [60,61], cancer initiation and metastasis [62]. miRNA-362-5p has been classified as oncogenic in solid tumors [63] and could be a potential therapeutic target or prognostic factor for human cancers [63]. In gastric cancer, it is upregulated, displaying its oncogenic function by inhibiting tumor suppression cylindromatosis [63]. Its downregulation was reported in cervical cancer promotes vascular invasion and metastasis [64] miRNA-363-3p is well known as miRNA with an anti-tumor role in many human cancers such as hepatocellular carcinoma and lung cancer [65,66]. It blocks cell proliferation, migration and invasion [65]. miRNA-363-3p downregulation correlates with metastasis in colorectal and hepatocellular cancers [67]. miRNA-204-5p is an example of oncogenic miRNA with dual function [68]. In solid tumors it mainly acts as a tumor suppressor (e.g., breast, prostate cancers and metastatic lung cancer [69] and in colorectal cancer was described as an inhibitor of proliferation and promotes cancer sensitivity to chemotherapy [70]. In contrast, miRNA-204-5p has been found upregulated in leukemia, although its role in disease development is unknown [71].

Targets of selected miRNAs identified in ccRCC tumors are involved in similar pathways like in other cancer types. It is worth to mention that miRNA-210-3p, which targets are involved in oxygen metabolism (Table 1, Table S3 and Figure S4b), is upregulated in both ccRCC and pRCC tumors but not in oncocytoma (Figure 2b), where lack of hypoxia and HIF1 stabilization are documented [72,73]. Additionally, patients with high level of miRNA-210 have lower risk for death (Figure 5b). Other interesting examples are potential targets of miRNA-362-3p belong to homotypic fusion and vacuole protein sorting (HOPS) complex (Table 1, Table S3 and Figure S4a), controlling cell homeostasis, which dysfunctions are associated with various cancer types including renal cancers [74]. miRNA-363-3p targets stand out from other miRNA target genes since they are involved in post-transcriptional control of RNA metabolism (Table 1, Table S3 and Figure S4a).

Deregulation of analyzed miRNA in ccRCC could be caused by different processes. For example, upregulation of miR-210-3p in ccRCC is caused by the elevated level of HIF [75]. In general, the expression of host genes correlates with miRNA expression, which suggests that deregulation is linked to transcription (Figure S3b,c). Additionally, miRNA expression could be regulated during processing exemplified by the decreasing percentage of longer isoforms (e.g., miRNA-363-3p) (Figure 4a–d), suggestive of effective degradation after maturation. Furthermore, adenylation is a well-known mechanism of miRNA-21-5p destabilization [76]. Reduction of adenylated miRNA-21-5p isoforms in ccRCC suggests its stabilization which could act together with high transcription efficiency and explain the miRNA-21-5p increased level in ccRCC (Figure 4a). Changes in iso-miRNA tailing are potentially related to upregulation of *TUT4* and *PAPD4*, factors involved in post-maturation miRNA modifications (Figure 4h). Further investigation of the reasons of miRNA deregulation in ccRCC is needed, especially when miRNAs are considered as therapeutic targets. Potential therapeutics could inhibit or mimic mature deregulated miRNAs although regulation of their expression by targeting of the mentioned above process is also possible [77].

As a result of our meta-analysis, we would like to propose a miRNA panel that could potentially aid RCC classification, with miRNA-362-5p, miRNA-363-3p, miRNA-224-5p, miRNA-155-5p and miRNA-210-3p as classifiers of ccRCC, miRNA-362-5p, miRNA-363-3p, miRNA-21-5p, miRNA-204-5p as characteristic for pRCC and miRNA-204-5p and miRNA-224-5p for oncocytoma (Figure 1, Figure 2 and Figure 3). Interestingly, our data suggest that miRNA-155-5p is a promising ccRCC-specific miRNA because it is unchanged in pRCC and oncocytoma (Figure 2b and Figure 3) and is highly upregulated in all ccRCC tumor regardless Fuhrman grade (Figure 2f and Figure 3). It was shown previously that the elevated level of this miRNA correlates with poor ccRCC outcome [78]. Additionally, our results suggest that analysis of specific miRNA isoforms (Figure 4) could increase the number of tested molecules in potential miRNA panel. Combination of the expression pattern of the seven mentioned above miRNAs is likely to be an indicative of the RCC tumor subtype. The most downregulated miRNA-200c in ccRCC seems to be a poor biomarker candidate. Regrettably, we did not validate the alteration of miRNA-200c-3p expression in ccRCC tumors likely due to low specificity of the qPCR primer. According to all NGS experiments, miRNA-200 family comprises a low percentage of miRNA-200c-3p in the kidney, therefore our results can be explained by amplification of the remaining family members (Figure S2). Unfortunately, data from high-throughput analysis of miRNA in ccpRCC and tcRCC with adjacent control tissue are not available and we did not include these RCC subtypes into the meta-analysis. However, ccpRCC and tcRCC could be mistaken with ccRCC and pRCC, respectively, during the diagnosis [7,8]. Both RCC subtypes show unique miRNA expression patterns distinguish them from the other RCCs [79,80], hence including these subtypes in meta-analysis should be considerate in the future.

A number of above-mentioned miRNAs might be useful to differentiate ccRCC tumor grading and such approach is poorly represented in the literature. For example, miRNA-224-5p is significantly upregulated in ccRCC first grade and oncocytoma (Figure 2b,f) however, its longer isoforms are significantly downregulated in ccRCC G4 (Figure 4f). Simultaneously, miRNA-21-5p is upregulated in ccRCC G4 (Figure 2f) and in pRCC (Figure 2b). Likewise, miRNA-210-3p is upregulated in ccRCC and pRCC, with no change in oncocytoma (Figure 2b). If status of these three miRNAs and its isoforms (Figure 4e–g) would be reflected in liquid biopsies or core needle biopsies [81] (non-invasive or less invasive biopsy methods than nephrectomy, respectively) it is worth to considerate their combination as ccRCC tumor grade indicator or panel distinguishing benign and malignant renal tumors (Figure 3). This is important for oncocytoma cases where surgical intervention is usually not required [82].

Although promising, suggested miRNA panel (Figure 3) requires additional validation in an independent sample set, in analyses additionally taking under consideration the heterogeneity of the tumor. Moreover, it would be interesting to test if proposed miRNA panel would be useful as a non-invasive classification test utilizing plasma and urine from RCC patients, as was shown previously that miRNA-210-3p was found circulating in the serum of mice tumors with hypoxia [83].

In conclusion, based on the meta-analysis and qPCR confirmation we propose panel of six miRNAs, with potential to distinguish ccRCC tumor grades (if extended with isoform analysis) and between RCC subtypes, which if validated further, may aid RCC classification in the future.

## 5. Conclusions

In conclusion, we found eight miRNAs to be commonly deregulated in ccRCC tumors; additionally, their levels can be used to distinguish RCC subtypes. Functions of these miRNAs have a potential impact on ccRCC etiology and/or development. Our results revealed that changes in the expression of selected miRNA might be potentially utilized as a tool aiding ccRCC subclass discrimination and we propose a miRNA panel that could be potentially utilized as a tool for RCC subtype distinction.

## Figures and Tables

**Table 1:** Gene ontology (GO) terms for targets of commonly deregulated miRNA in ccRCC.

GO Id	Description	miRNA Targets
GO:0038093	Fc receptor signaling pathway	miR-200c-3p, miR-224-5p, miR-155-5p
GO:0002768	immune response-regulating cell surface receptor signaling pathway	miR-200c-3p, miR-204-5p, miR-224-5p
GO:0038179	neurotrophin signaling pathway	miR-200c-3p, miR-224-5p, miR-155-5p
GO:0071774	response to fibroblast growth factor	miR-200c-3p, miR-224-5p
GO:0030897	HOPS complex	miR-362-5p
GO:0000289	nuclear-transcribed mRNA poly(A) tail shortening	miR-363-3p
GO:0007178	transmembrane receptor protein serine/threonine kinase signaling pathway	miR-204-5p
GO:0071559	response to transforming growth factor beta	miR-204-5p, miR-155-5p
GO:0071214	cellular response to abiotic stimulus	miR-21-5p
GO:0034142	toll-like receptor 4 signaling pathway	miR-21-5p
GO:0019787	small conjugating protein ligase activity	miR-21-5p
GO:0019901	protein kinase binding	miR-155-5p
GO:0051169	nuclear transport	miR-155-5p
GO:0010608	posttranscriptional regulation of gene expression	miR-155-5p
GO:0071456	cellular response to hypoxia	miR-210-3p
GO:1901989	positive regulation of cell cycle phase transition	miR-210-3p
GO:0010639	negative regulation of organelle organization	miR-210-3p
GO:0007059	chromosome segregation	miR-210-3p

## Data Availability

Publicly available datasets were analyzed in this study. This data can be found here: https://www.ncbi.nlm.nih.gov/geo/query/acc.cgi?acc=GSE151428.

## Supplementary Materials

The following are available online at https://www.mdpi.com/2072-6694/13/3/548/s1, Figure S1. The meta-analysis workflow. Figure S2. miRNA-200-3p family. Figure S3. Potential reasons of miRNA deregulation in ccRCC tumors. Figure S4. miRNA function in ccRCC tumor. Table S1. List of patients. Table S2. Identified iso-miRNAs in healthy and ccRCC kidney tissues in Exp1 and Exp4 data sets. The average percentage of miRNA isoforms. Table S3. Top 10 gene ontology (GO) terms for targets of commonly deregulated miRNA in ccRCC. Table S4. Primers used in the experimental procedures.
