# Supplementary material for: Identification of RCC Subtype-Specific microRNAs–Meta-Analysis of High-Throughput RCC Tumor microRNA Expression Data
Source: Cancers (Basel). 2021 Feb 1;13(3):548. doi: 10.3390/cancers13030548 (PMC7867039; doi:10.3390/cancers13030548)
Supplement: Supplementary file 1 [file cancers-13-00548-s001.zip › Supplementary_Figures.pdf]

cancers

MDPI

Supplementary Materials

Identification of RCC Subtype-Specific microRNAs–Meta-Analysis of High-Throughput RCC Tumor microRNA Expression Data

Arkadiusz Kajdasz, Weronika Majer, Katarzyna Kluzek, Jacek Sobkowiak, Tomasz Milecki, Natalia Derebecka, Zbigniew Kwias, Hans A.R. Bluyssen and Joanna Wesoly

(a)

No.	RCC subtype	Deregulated miRNAs		Samples		Experiment type	Ref.
		Down	Up	Ctrl	RCC		
Exp1	ccRCC	127	32	6	24	NGS	This work
Exp2	ccRCC	76	30	10	10	NGS	[28]
Exp3	ccRCC	44	42	11	15	NGS	[29]
Exp4	ccRCC	243	181	18	18	NGS	[30]
Exp5	ccRCC	165	233	71	512	NGS	TCGA (dbDEMC2.0)
Exp6	ccRCC	33	33	8	8	Microarray	[40]
Exp7	ccRCC	32	48	12	12	Microarray	[41]
Exp8	ccRCC	49	48	13	13	Microarray	[42]
Exp9	pRCC	153	245	34	290	NGS	TCGA (dbDEMC2.0)
Exp10	pRCC	35	42	4	4	Microarray	[40]
Exp11	pRCC	17	17	18	7	Microarray	[43]
Exp12	chRCC	171	180	25	66	NGS	TCGA (dbDEMC2.0)
Exp13	chRCC	12	6	4	4	Microarray	[40]
Exp14	onco.	14	20	20	14	Microarray	[40]

(b)

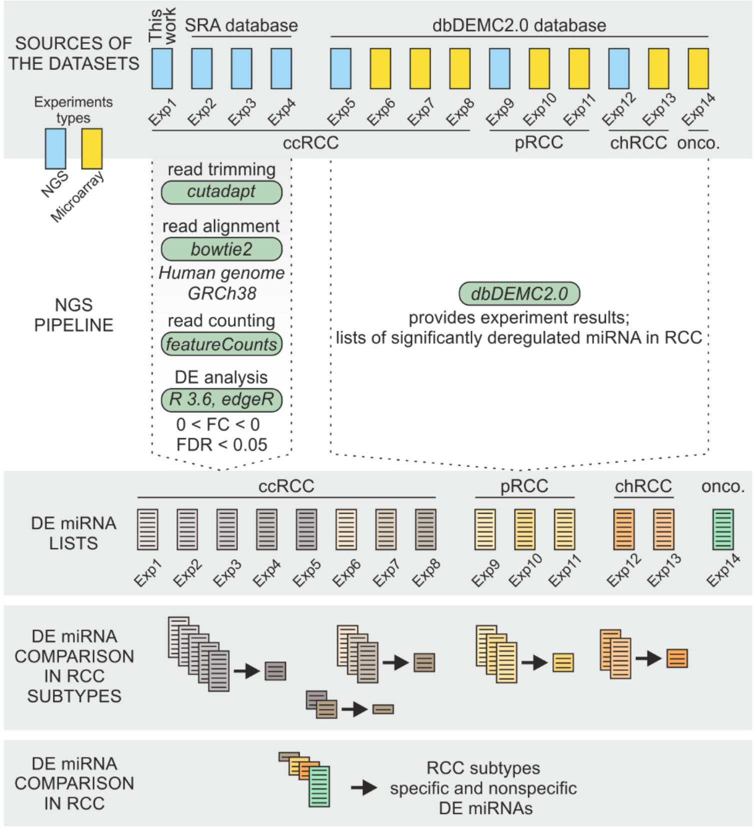

Figure S1. The meta-analysis workflow. (**a**) List of data sets used in the meta-analysis; (**b**) Analysis workflow. Raw NGS data was re-ana\lyzed using identical data processing pipeline. Microarray and The Cancer Genome Atlas (TCGA) results were obtained in a form of a list of deregulated miRNAs from the dbDEMC2.0 database. ccRCC, clear renal cell carcinoma; pRCC, papillary renal cell carcinoma; chRCC, chromophobe renal cell carcinoma; onco., oncocytoma; FDR, the false discovery rate; FC, fold change; DE, differential expression; Exp1-8, experiments 1-8.

(a)

(b)

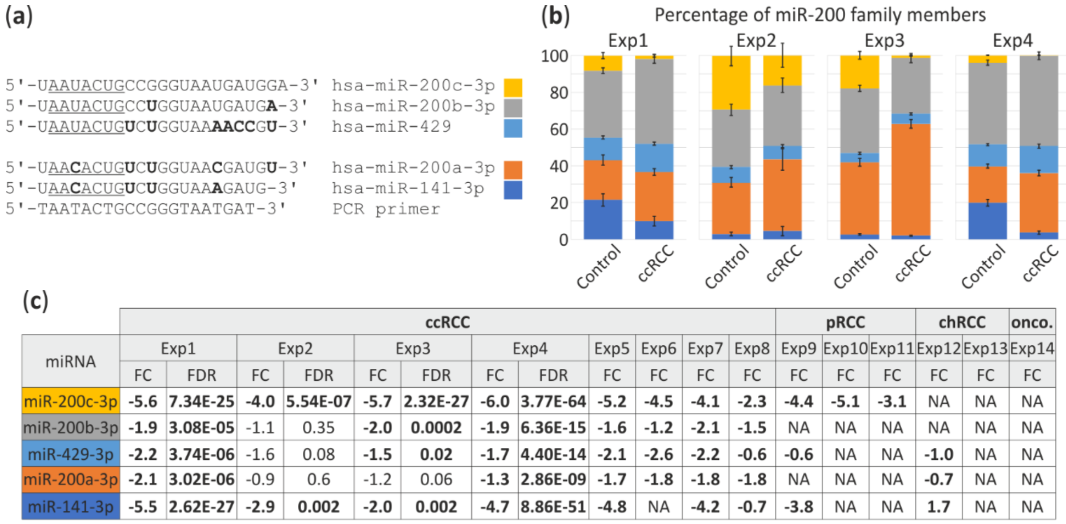

(c)

miRNA	ccRCC												pRCC			chRCC		onco.
	Exp1		Exp2		Exp3		Exp4		Exp5	Exp6	Exp7	Exp8	Exp9	Exp10	Exp11	Exp12	Exp13	Exp14
	FC	FDR	FC	FDR	FC	FDR	FC	FDR	FC	FC	FC	FC	FC	FC	FC	FC	FC	FC
miR-200c-3p	**-5.6**	**7.34E-25**	**-4.0**	**5.54E-07**	**-5.7**	**2.32E-27**	**-6.0**	**3.77E-64**	**-5.2**	**-4.5**	**-4.1**	**-2.3**	**-4.4**	**-5.1**	**-3.1**	NA	NA	NA
miR-200b-3p	**-1.9**	**3.08E-05**	-1.1	0.35	**-2.0**	**0.0002**	**-1.9**	**6.36E-15**	**-1.6**	**-1.2**	**-2.1**	**-1.5**	NA	NA	NA	NA	NA	NA
miR-429-3p	**-2.2**	**3.74E-06**	-1.6	0.08	**-1.5**	**0.02**	**-1.7**	**4.40E-14**	**-2.1**	**-2.6**	**-2.2**	**-0.6**	**-0.6**	NA	NA	**-1.0**	NA	NA
miR-200a-3p	**-2.1**	**3.02E-06**	-0.9	0.6	-1.2	0.06	**-1.3**	**2.86E-09**	**-1.7**	**-1.8**	**-1.8**	**-1.8**	NA	NA	NA	**-0.7**	NA	NA
miR-141-3p	**-5.5**	**2.62E-27**	**-2.9**	**0.002**	**-2.0**	**0.002**	**-4.7**	**8.86E-51**	**-4.8**	NA	**-4.2**	**-0.7**	**-3.8**	NA	NA	**1.7**	NA	NA

Figure S2. miRNA-200-3p family. (**a**) Sequence of miRNA-200-3p family members. Underline, seed sequence; bold, differences in miRNA sequences according to miRNA-200c-3p; (**b**) The percentage contribution of miRNA-200-3p family members in control and ccRCC tissues in NGS experiments; (**c**) Expression levels of miRNA-200-3p family members in studies used in the meta-analysis. Bold, significantly deregulated miRNA (FDR < 0.05). FC, fold change; FDR, the false discovery rate.

(a)

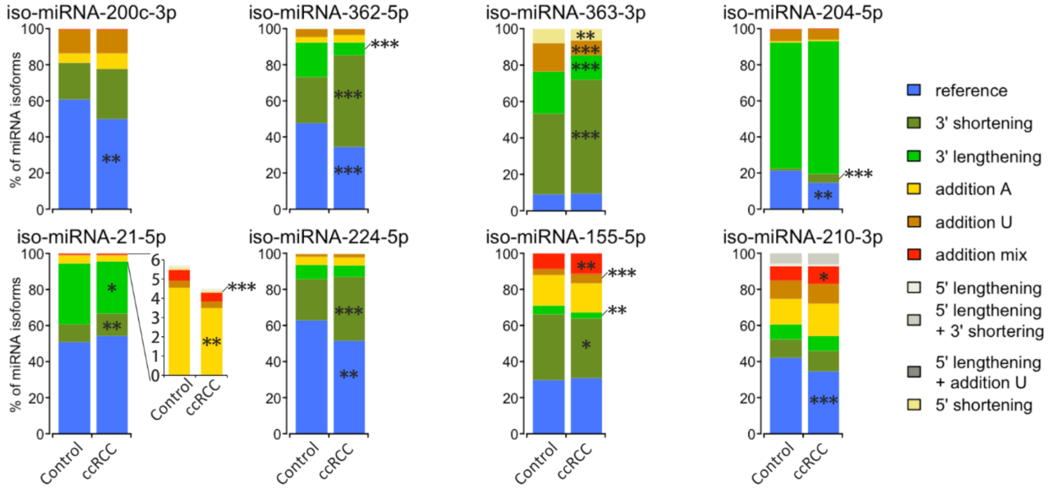

(b)

miRNA	host gene		
	name	log2 FC	FDR
miRNA-200c	*MIR200CHG*	-4.4	0.0002
miRNA-204	*TRPM3*	-3.1	5.68E-14
miRNA-362	*CLCN5*	-2.4	1.03E-11
miRNA-224	*GABRE*	0.9	0.04
miRNA-21	*VMP1*	1.0	0.0001
mIRNA-155	*MIR155HG*	3.3	1.19E-14
miRNA-210	*MIR210HG*	3.3	5.95E-09

(c)

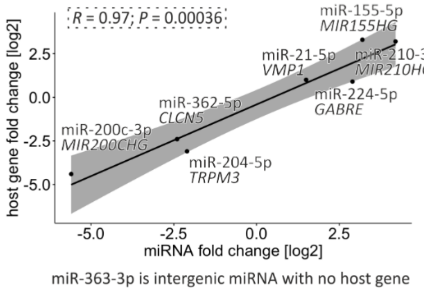

Figure S3. Potential reasons of miRNA deregulation in ccRCC tumors. (**a**) Isoforms of miRNA in ccRCC from Exp4 data set. Percentage of 8 commonly deregulated miRNA isoforms (iso-miRNA) in control and ccRCC tissues based on Exp4. Part of the chart represents the percentage of iso-miRNA-21-5p is magnified to show more accurately 3' tailing of miRNA-21-5p; (**b, c**) Co-transcriptional reason of miRNA deregulation in ccRCC. (**a**) Deregulation of host genes of 8 commonly deregulated miRNA in ccRCC based on RNA-Seq data. (**b**) Correlation of miRNA host genes and mature miRNA expression in ccRCC based on RNA-Seq and small RNA-Seq (Exp1) with exception of intergenic miRNA-363. FC, fold change; FDR, the false discovery rate.

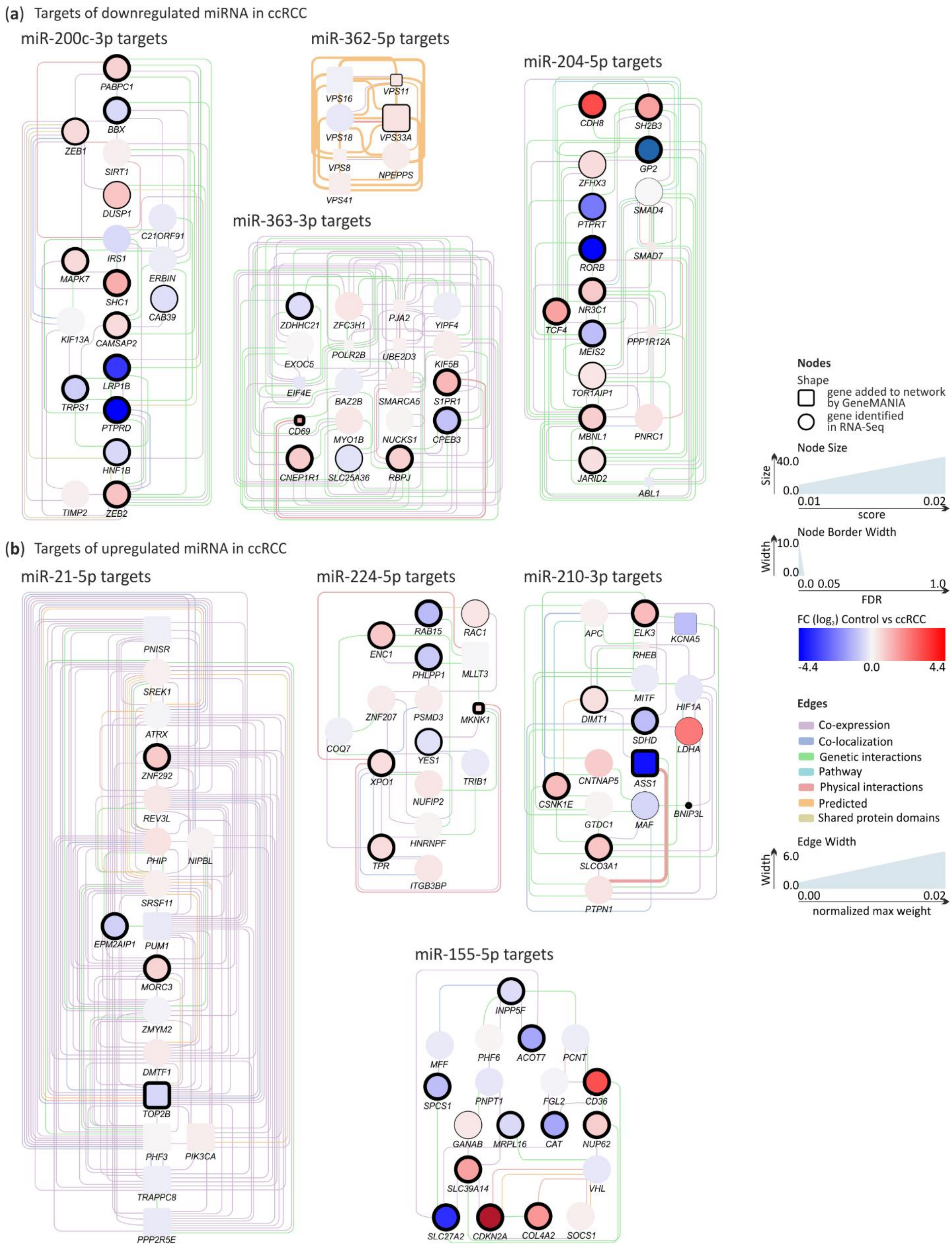

Figure S4. miRNA function in ccRCC tumor. The most probable clusters created with potential miRNA targets by GeneMANIA and MCODE. Clusters of targets of commonly downregulated (**a**) or upregulated (**b**) miRNA in ccRCC. Nodes, genes with expression level and FDR values based on RNA-Seq in ccRCC tumors; circle, potential miRNA targets (query genes); square, genes added by GeneMANIA to the network. Node size score, prediction of the genes relation provided by GeneMANIA; edge, line links two nodes, color and thickness represent kind and weight of the interaction, respectively.
