# Supplementary material for: Identification of RCC Subtype-Specific microRNAs–Meta-Analysis of High-Throughput RCC Tumor microRNA Expression Data
Source: Cancers (Basel). 2021 Feb 1;13(3):548. doi: 10.3390/cancers13030548 (PMC7867039; doi:10.3390/cancers13030548)
Supplement: Supplementary file 1 [file cancers-13-00548-s001.zip › Supplementary_Tables.pdf]

Supplementary Table S1. List of patients.

Patient name	RCC subtype	Gender	TNM	Metastasis	Fuhrman grade	Control tissue NGS	Control tissue qPCR	Analysis in which sample were used
001C	ccRCC	M	3	N	3			RNA-Seq
003S	ccRCC	F	4	Y	4			miRNA-Seq
004C	ccRCC	M	3	N	3			RNA-Seq, miRNA-Seq
005C	ccRCC	F	1	N	3			RNA-Seq
006C	ccRCC	M	3	N	4			RNA-Seq
009C	ccRCC	M	3	N	3			RNA-Seq
011C	ccRCC	F	3	N	2			RNA-Seq
012C	ccRCC	M	4	Y	4			RNA-Seq, miRNA-Seq
016C	ccRCC	F	1	N	2			RNA-Seq, miRNA-Seq
019C	ccRCC	M	1	N	2			RNA-Seq, miRNA-Seq
024C	ccRCC	M	3	Y	2			RNA-Seq, miRNA-Seq
028C	ccRCC	M	1	N	2			RNA-Seq
030C	ccRCC	M	1	N	2			RNA-Seq
034C	ccRCC	M	1	N	2			RNA-Seq
035C	ccRCC	M	1	N	3			RNA-Seq
036C	ccRCC	F	3	N	3			miRNA-Seq
045C	ccRCC	M	3	N	4			RNA-Seq, miRNA-Seq
047C	ccRCC	M	1	N	2			miRNA-Seq
48C	ccRCC	F	1		2			miR-qPCR, mRNA-qPCR, iso-qPCR
050C	ccRCC	F	3	Y	2			RNA-Seq, miRNA-Seq, miR-qPCR, mRNA-qPCR, iso-qPCR
058C	ccRCC	F	3	N	3			miRNA-Seq, miR-qPCR, mRNA-qPCR, iso-qPCR
059C	ccRCC	F	1	Y	2			RNA-Seq, miRNA-Seq
062C	ccRCC	F	1	N	3			RNA-Seq
065C	ccRCC	F	1	N	1			RNA-Seq, miR-qPCR, mRNA-qPCR, iso-qPCR
067C	ccRCC	F	3	Y	2			RNA-Seq, miRNA-Seq
068C	ccRCC	F	3	Y	2			RNA-Seq, miRNA-Seq
069C	ccRCC	F	1	N	2			RNA-Seq
72C	ccRCC		1		4			miR-qPCR, mRNA-qPCR, iso-qPCR
073C	ccRCC	F	1	N	2			RNA-Seq
074C	ccRCC	M	1	N	1			RNA-Seq
075C	ccRCC	F	2	N	2			RNA-Seq
081C	ccRCC	F	1	N	1			RNA-Seq, miR-qPCR, mRNA-qPCR, iso-qPCR
082C	ccRCC	F	1	N	2			RNA-Seq
083C	ccRCC	M	3	Y	3			RNA-Seq, miRNA-Seq, miR-qPCR, iso-qPCR
084C	ccRCC	F	3	N	4			RNA-Seq, miRNA-Seq, miR-qPCR, mRNA-qPCR, iso-qPCR
090C	ccRCC	F	1	N	2			RNA-Seq
091C	ccRCC	F	3	N	3	Y		RNA-Seq
096C	ccRCC	M	1	N	4			RNA-Seq, miRNA-Seq, iso-qPCR
101	ccRCC	M	3		2			miR-qPCR
103	ccRCC	F	3		1			miR-qPCR, mRNA-qPCR, iso-qPCR
104C	ccRCC	F	3	N	3			RNA-Seq, miRNA-Seq
106C	ccRCC	F	3	N	3	Y		RNA-Seq
107C	ccRCC	F	1	N	1			RNA-Seq

110	ccRCC	F	3		4			miR-qPCR, iso-qPCR
115	ccRCC	F	1		2			miR-qPCR
117C	ccRCC	M	3	N	3			RNA-Seq
118C	ccRCC	F	3	Y	3			RNA-Seq, miRNA-Seq
119C	ccRCC	M	3	N	4	Y		RNA-Seq, miR-qPCR, mRNA-qPCR
120C	ccRCC	M		N	2			miRNA-Seq
123C	ccRCC	F	1	N	3	Y		RNA-Seq, miRNA-Seq
124C	ccRCC	F	1	N	2			RNA-Seq
125C	ccRCC	F	3	N	3			RNA-Seq
126C	ccRCC	M	1	N	2	Y		RNA-Seq
131C	ccRCC	M	3		2			miR-qPCR, mRNA-qPCR, iso-qPCR
132C	ccRCC	M	1	N	3	Y	Y	RNA-Seq, miR-qPCR, mRNA-qPCR, iso-qPCR
134	ccRCC	M		N	3			miRNA-Seq
143C	ccRCC	M	3	Y	2	Y	Y	RNA-Seq, miR-qPCR
147C	ccRCC	M	3	N	2	Y		RNA-Seq
149C	ccRCC	M	1	y	3	Y		RNA-Seq, miRNA-Seq
153C	ccRCC	M	3	Y	3	Y		RNA-Seq, miRNA-Seq
156	ccRCC				3	Y		RNA-Seq
158C	ccRCC	F	3	N	3	Y		RNA-Seq
162C	ccRCC	M	1	N	3			RNA-Seq
164C	ccRCC	F	2	Y	4			RNA-Seq
169C	ccRCC	M	3	N	4			RNA-Seq
171B	ccRCC	F	1	N	2	Y		RNA-Seq
172B	ccRCC	F	3	N	2			RNA-Seq, miRNA-Seq
175C	ccRCC	F	1	N	3			RNA-Seq
178C	ccRCC	M	3	N	3			RNA-Seq, miRNA-Seq
179C	ccRCC	M	3	N	2	Y		RNA-Seq, miRNA-Seq
180C	ccRCC	M	1	N	2	Y		RNA-Seq, miRNA-Seq
182C	ccRCC	M	3	N	3	Y		RNA-Seq
183C	ccRCC	M	3	Y	4	Y		RNA-Seq
185C	ccRCC	F	3	Y	3			RNA-Seq, miR-qPCR, mRNA-qPCR, iso-qPCR
217	ccRCC	M	3		3			miR-qPCR
220c	ccRCC	M	3		4		Y	miR-qPCR, mRNA-qPCR, iso-qPCR
221c	ccRCC	M	1		1			miR-qPCR
222C	ccRCC	F	1		1			miR-qPCR
225c	ccRCC	M	3		3		Y	miR-qPCR
227c	ccRCC	F	2		4		Y	miR-qPCR
229c	ccRCC	M	1		1			miR-qPCR
241c	ccRCC	F	2		2			miR-qPCR
271C	ccRCC	M	1		2			miR-qPCR
291c	ccRCC		3		2		Y	miR-qPCR
293C-1	ccRCC	M	1		1		Y	miR-qPCR, mRNA-qPCR, iso-qPCR
294	ccRCC	F	1		1		Y	miR-qPCR, mRNA-qPCR
299c	ccRCC	M	1		1		Y	miR-qPCR
302c	ccRCC	M	1		3		Y	miR-qPCR, mRNA-qPCR, iso-qPCR
303c	ccRCC	M	3		3			miR-qPCR
306c	ccRCC	F	2		1			miR-qPCR
308	ccRCC	M	1		4			miR-qPCR

316c	ccRCC	M	2		4		Y	miR-qPCR
318C	ccRCC		3		3		Y	miR-qPCR
319c	ccRCC	M	1		4		Y	miR-qPCR
S10c	ccRCC	M	3		2			miR-qPCR
s19c	ccRCC	M	1		3		Y	miR-qPCR
s8C	ccRCC	M	3		2		Y	miR-qPCR
77C	onco.							miR-qPCR
78C	onco.							miR-qPCR
210	onco.	M						miR-qPCR
s18c	onco.	F						miR-qPCR
s26c	onco.	M						miR-qPCR
236	onco.	M						miR-qPCR
208	onco.	M						miR-qPCR
228	onco.	M						miR-qPCR
010C	pRCC							miR-qPCR
013C	pRCC							miR-qPCR
029C	pRCC							miR-qPCR
33C	pRCC							miR-qPCR
39C	pRCC							miR-qPCR
51C	pRCC							miR-qPCR
272c	pRCC	M					Y	miR-qPCR
288c	pRCC	M					Y	miR-qPCR
311c	pRCC						Y	miR-qPCR
322c	pRCC	F					Y	miR-qPCR
120	only control	M	3			Y	Y	miR-qPCR
135	only control						Y	miR-qPCR

ccRCC, clear cell renal cell carcinoma; pRCC, papillary renal cell carcinoma; onco., oncocytoma; F, female; M, male; TNM, TNM Classification of Malignant Tumors; samples usage: miR-qPCR, qPCR for miRNA; iso-qPCR, qPCR for isoforms of miRNA; mRNA-qPCR, qPCR for mRNA

Supplementary Table S2. Identified iso-miRNAs in healthy and ccRCC kidney tissues in Exp1 and Exp4 data sets. The average percentage of miRNA isoforms.

	Exp1				Exp4			
	AVG ccRCC [%] n = 26	AVG healthy [%] n = 6	Ratio in ccRCC	*P*-value	AVG ccRCC [%] n = 18	AVG healthy [%] n = 18	Ratio in ccRCC	*P*-value
**hsa-miR-200c-3p**								
5′-CGGUUGGGAGUCUCUAAUACUGCCGGGUAAUGAUGGAGG-3′								
reference UAAUACUGCCGGGUAAUGAUGGA	65.0667	63.2316	1.0290	0.8926	50.0482	60.7906	0.8233	**0.0029**
UAAUACUGCCGGGUAAUGAUGG	21.2633	26.6606	0.7976	0.6307	21.5313	17.8480	1.2064	0.2040
UAAUACUGCCGGGUAAUGAUGGAU	1.7062	2.3420	0.7285	0.7871	2.9506	2.9663	0.9947	0.9911
UAAUACUGCCGGGUAAUGAUGGU	11.9638	7.7658	1.5406	0.6636	8.7295	9.8789	0.8837	0.5968
UAAUACUGCCGGGUAAUGAUG	NA	NA	NA	NA	0.5779	1.2904	0.4478	0.1505
UAAUACUGCCGGGUAAUGAU	NA	NA	NA	NA	5.5160	1.1923	4.6266	0.0581
UAAUACUGCCGGGUAAUGAUGGAA	NA	NA	NA	NA	7.5810	4.3595	1.7389	0.1721
UAAUACUGCCGGGUAAUGAUGA	NA	NA	NA	NA	1.0053	0.4701	2.1383	0.3915
UAAUACUGCCGGGUAAUGAUGAA	NA	NA	NA	NA	0.1852	0.2383	0.7771	0.7859
UAAUACUGCCGGGUAAUGAUGGUA	NA	NA	NA	NA	0.4657	0.3657	1.2734	0.8179
UAAUACUGCCGGGUAAUGAUGGUU	NA	NA	NA	NA	1.4095	0.5999	2.3496	0.3544
**has-miRNA-362-5p**								
5′-UGAAUCCUUGGAACCUAGGUGUGAGUGCUAUUUCAGUGCA-3′								
AAUCCUUGGAACCUAGGUGUGAGU reference	63.3643	64.1196	0.9882	0.9167	34.4809	47.4512	0.7267	**0.0001**
AAUCCUUGGAACCUAGGUGUGAGUGC	5.5141	5.8861	0.9368	0.8880	5.1825	17.5568	0.2952	**0.0000**
AAUCCUUGGAACCUAGGUGUGA	3.7886	3.3526	1.1300	0.8680	7.6758	4.1270	1.8599	**0.0316**
AAUCCUUGGAACCUAGGUGUGAG	22.7541	24.4667	0.9300	0.7620	42.9575	21.4470	2.0030	**0.0000**
AAUCCUUGGAACCUAGGUGUGAGA	4.5789	2.1749	2.1054	0.4572	4.3160	2.9306	1.4727	0.2297
AAUCCUUGGAACCUAGGUGUGAGUG	NA	NA	NA	NA	2.0240	1.9266	1.0506	0.8990
AAUCCUUGGAACCUAGGUGUGAGUGU	NA	NA	NA	NA	3.3632	4.5607	0.7374	0.5139
**has-miRNA-363-3p**								
5′-UCAUAGGAGAAAAAUUGCACGGUAUCCAUCUGUAAACC-3′								
reference AAUUGCACGGUAUCCAUCUGUA	6.8393	8.7565	0.7810	0.1467	9.3545	9.0175	1.0374	0.5859
AAUUGCACGGUAUCCAUCUGU	40.0607	41.6244	0.9624	0.6445	43.7802	38.6888	1.1316	**0.0206**
AAUUGCACGGUAUCCAUCUGUAA	13.8762	20.1272	0.6894	**0.0467**	13.6252	22.7559	0.5988	**0.0000**
AAUUGCACGGUAUCCAUCUG	26.6324	9.9151	2.6860	**0.0057**	18.7404	5.7392	3.2653	**0.0000**
AUUGCACGGUAUCCAUCUGU	3.2057	2.1532	1.4888	0.2307	2.4178	1.5468	1.5631	**0.0006**
AUUGCACGGUAUCCAUCUGUAA	2.0746	2.7705	0.7488	0.3825	2.5013	3.6281	0.6894	**0.0006**
AAUUGCACGGUAUCCAUCUGUAU	6.0911	12.0411	0.5059	**0.0018**	7.8480	15.6874	0.5003	**0.0000**
AUUGCACGGUAUCCAUCUGUAU	1.2200	2.6119	0.4671	**0.0256**	1.7326	2.9362	0.5901	**0.0001**
**hsa-miR-204-5p**								
5′-GACUUCCCUUUGUCAUCCUAUGCCUGAGAAUAUAUGAAGG-3′								
UUCCCUUUGUCAUCCUAUGCCU reference	56.3051	41.9514	1.3422	0.0704	14,6926	21,2192	0,6924	**0,0023**
UUCCCUUUGUCAUCCUAUG	3.6779	1.5841	2.3218	0.2224	2,4267	0,2528	9,5981	**0,0001**
UUCCCUUUGUCAUCCUAUGCCUG	27.9791	48.5108	0.5768	**0.0007**	73,1006	68,9891	1,0596	0,0877
UUCCCUUUGUCAUCCUAUGCCUGA	0.5211	0.5341	0.9757	0.9748	0,8670	0,8514	1,0183	0,8263
UUCCCUUUGUCAUCCUAUGCC	4.7758	2.1122	2.2611	**0.0112**	1,8751	0,8914	2,1036	**0,0020**
UUCCCUUUGUCAUCCUAUGCCUA	0.3268	0.4086	0.7998	0.4680	0,2492	0,3119	0,7989	0,0829
UUCCCUUUGUCAUCCUAUGCCA	1.2556	0.8033	1.5631	0.2513	0,5209	0,4065	1,2815	0,1685
UUCCCUUUGUCAUCCUAUGCCUU	0.6676	0.7279	0.9171	0.8198	0,5806	0,7603	0,7637	**0,0065**
UUCCCUUUGUCAUCCUAUGCCUGU	4.4911	3.3676	1.3336	0.7768	5,0199	5,6498	0,8885	0,1422

UUCCCUUUGUCAUCCUAUGC	NA	NA	NA	NA	0,2766	0,0912	3,0337	**0,0000**
UUCCCUUUGUCAUCCUAUGCAU	NA	NA	NA	NA	0,1301	0,2283	0,5699	**0,0091**
UUCCCUUUGUCAUCCUAUGCCUGUA	NA	NA	NA	NA	0,1511	0,1583	0,9546	0,7671
UUCCCUUUGUCAUCCUAUGCCUUU	NA	NA	NA	NA	0,0415	0,0869	0,4770	**0,0001**
UUCCCUUUGUCAUCCUAUGCCUGUU	NA	NA	NA	NA	0,0681	0,1028	0,6622	0,0254
**hsa-miRNA-21-5p**								
5′-GGUAGCUUAUCAGACUGAUGUUGACUGUUGAAUCUCAUGG-3′								
UAGCUUAUCAGACUGAUGUUGA reference	53.14	51.95	1.023	0.578	53.9763	50.6859	1.0649	0.0807
UAGCUUAUCAGACUGAUGUUG	12.01	12.18	0.985	0.8889	10.8748	8.8147	1.2337	**0.0065**
UAGCUUAUCAGACUGAUGUUGAC	16.74	14.94	1.12	0.2806	14.3796	15.3866	0.9346	0.3699
UAGCUUAUCAGACUGAUGUUGACU	12.37	14.29	0.866	0.2381	14.6200	18.2979	0.7990	**0.0082**
UAGCUUAUCAGACUGAUGUU	1.964	1.854	1.059	0.7446	1.4899	0.9701	1.5357	**0.0000**
UAGCUUAUCAGACUGAUGU	0.643	0.654	0.984	0.9327	0.2533	0.2239	1.1317	0.1539
UAGCUUAUCAGACUGAUGUUGAA	1.016	1.236	0.822	**0.0257**	1.1578	1.3301	0.8704	0.0833
UAGCUUAUCAGACUGAUGUUGACA	1.654	2.227	0.743	**0.0005**	1.6658	2.4824	0.6710	**0.0002**
UAGCUUAUCAGACUGAUGUUA	0.103	0.118	0.871	0.5089	0.1212	0.0875	1.3857	**0.0337**
UAGCUUAUCAGACUGAUGUUGAAA	0.171	0.278	0.617	**0.0036**	0.3742	0.4875	0.7674	**0.0420**
UAGCUUAUCAGACUGAUGUUGAU	0.109	0.143	0.764	0.0856	0.2693	0.3542	0.7603	**0.0316**
UAGCUUAUCAGACUGAUGUUGU	0.085	0.13	0.652	**0.0063**	0.2195	0.2406	0.9120	0.3706
GUAGCUUAUCAGACUGAUGUUGA	NA	NA	NA	NA	0.0620	0.0729	0.8506	0.1755
GUAGCUUAUCAGACUGAUGUUG	NA	NA	NA	NA	0.0251	0.0291	0.8609	0.2792
GUAGCUUAUCAGACUGAUGUUGAC	NA	NA	NA	NA	0.0158	0.0194	0.8170	0.4576
AGCUUAUCAGACUGAUGUUGA	NA	NA	NA	NA	0.0333	0.0361	0.9226	0.4627
AGCUUAUCAGACUGAUGUUGACU	NA	NA	NA	NA	0.0512	0.0766	0.6687	**0.0001**
UAGCUUAUCAGACUGAUGUA	NA	NA	NA	NA	0.0553	0.0372	1.4872	**0.0186**
UAGCUUAUCAGACUGAUGUUAA	NA	NA	NA	NA	0.0430	0.0407	1.0552	0.7380
UAGCUUAUCAGACUGAUGUAA	NA	NA	NA	NA	0.0133	0.0080	1.6526	**0.0134**
UAGCUUAUCAGACUGAUGUUGAAU	NA	NA	NA	NA	0.2693	0.3542	0.7603	**0.0316**
UAGCUUAUCAGACUGAUGUUU	NA	NA	NA	NA	0.0269	0.0288	0.9321	0.7274
UAGCUUAUCAGACUGAUGUUGAUA	NA	NA	NA	NA	0.0332	0.0518	0.6413	0.0777
UAGCUUAUCAGACUGAUGUUUA	NA	NA	NA	NA	0.1404	0.1497	0.9377	0.7545
**hsa-miR-224-5p**								
5′-CUUUCAAGUCACUAGUGGUUCCGUUUAGUAGAUGAUUGUG-3′								
UCAAGUCACUAGUGGUUCCGUUUAG reference	NA	NA	NA	NA	NA	NA	NA	NA
CAAGUCACUAGUGGUUCCGUUUAG	43.0228	55.4497	0.7759	0.1068	51.4793	62.6154	0.8222	**0.0013**
CAAGUCACUAGUGGUUCCGUUU	39.4255	16.2037	2.4331	**0.0022**	28.6966	17.0448	1.6836	**0.0001**
CAAGUCACUAGUGGUUCCGUUUA	7.9281	13.0026	0.6097	0.1417	5.7794	5.1270	1.1272	0.4465
CAAGUCACUAGUGGUUCCGUUUAGU	7.3273	14.2328	0.5148	0.1171	6.1545	8.1010	0.7597	0.1152
CAAGUCACUAGUGGUUCCGUU	2.2963	1.1111	2.0666	0.4019	1.0007	0.7514	1.3317	0.5151
UCAAGUCACUAGUGGUUCCGUUU	NA	NA	NA	NA	0.2779	0.4370	0.6358	0.4525
CAAGUCACUAGUGGUUCCGUUUAGA	NA	NA	NA	NA	1.5150	1.8239	0.8306	0.4761
CAAGUCACUAGUGGUUCCGUUUAA	NA	NA	NA	NA	1.8503	1.6890	1.0955	0.6789
CAAGUCACUAGUGGUUCCGUUA	NA	NA	NA	NA	0.9659	0.7651	1.2624	0.4632
CAAGUCACUAGUGGUUCCGUUAA	NA	NA	NA	NA	0.3588	0.0265	13.5616	**0.0147**
CAAGUCACUAGUGGUUCCGUUUU	NA	NA	NA	NA	1.0787	0.2562	4.2108	**0.0000**
CAAGUCACUAGUGGUUCCGUUUAU	NA	NA	NA	NA	0.6436	1.2121	0.5310	0.3882
CAAGUCACUAGUGGUUCCGUUUAGUU	NA	NA	NA	NA	0.1994	0.1507	1.3229	0.6331
**hsa-miR-155-5p**								
5′-CUGUUAAUGCUAAUCGUGAUAGGGGUUUUUGCCUCCAACU-3′								

UUAAUGCUAAUCGUGAUAGGGGUU reference	23.8234	21.3017	1.1184	0.3897	30.9644	29.6181	1.0455	0.4852
UUAAUGCUAAUCGUGAUAGGG	3.9100	7.6225	0.5130	**0.0003**	0.8104	1.0658	0.7603	0.1102
UUAAUGCUAAUCGUGAUAGGGGU	35.8693	34.3216	1.0451	0.5640	30.0607	32.1442	0.9352	0.1589
UUAAUGCUAAUCGUGAUAGGGGUUU	3.9968	4.1326	0.9672	0.8837	2.6426	3.8704	0.6828	**0.0035**
UUAAUGCUAAUCGUGAUAGGGG	6.4934	6.6740	0.9729	0.8112	2.0383	3.2344	0.6302	**0.0005**
UUAAUGCUAAUCGUGAUAGGGA	11.7495	11.0977	1.0587	0.7505	5.4075	5.5154	0.9804	0.8871
UUAAUGCUAAUCGUGAUAGGGGA	7.2448	7.0167	1.0325	0.8258	3.9582	5.4923	0.7207	**0.0134**
UUAAUGCUAAUCGUGAUAGGGAU	3.3309	5.0086	0.6650	0.0778	5.7477	4.1797	1.3751	**0.0364**
UUAAUGCUAAUCGUGAUAGGGU	3.5819	2.8248	1.2680	0.3280	4.5672	2.6639	1.7145	**0.0007**
UUAAUGCUAAUCGUGAUAGGGGUUUU	NA	NA	NA	NA	0.8925	1.1141	0.8010	0.2757
UUAAUGCUAAUCGUGAUAGGGGUUA	NA	NA	NA	NA	2.8899	2.6070	1.1085	0.5183
UUAAUGCUAAUCGUGAUAGGGGUA	NA	NA	NA	NA	2.6533	2.6253	1.0106	0.9256
UUAAUGCUAAUCGUGAUAGGGAA	NA	NA	NA	NA	1.1022	0.7556	1.4588	**0.0479**
UUAAUGCUAAUCGUGAUAGGGGUUAAU	NA	NA	NA	NA	0.8701	0.6706	1.2974	0.3805
UUAAUGCUAAUCGUGAUAGGGGUAU	NA	NA	NA	NA	0.5801	0.6879	0.8433	0.5183
UUAAUGCUAAUCGUGAUAGGGGAU	NA	NA	NA	NA	4.0374	3.3516	1.2046	0.0807
UUAAUGCUAAUCGUGAUAGGGUU	NA	NA	NA	NA	0.7777	0.4036	1.9268	**0.0025**
**hsa-miRNA-210-3p**								
5’-UGCCCCAGACCCACUGUGCGUGUGACAGCGGCUGAUCUG-3’								
reference CUGUGCGUGUGACAGCGGCUGA	42.8910	49.5276	0.8660	**0.0149**	34.4094	42.1745	0.8159	**0.0000**
CUGUGCGUGUGACAGCGGCUG	2.5423	1.9755	1.2869	0.3093	1.7822	2.0970	0.8499	0.5244
CUGUGCGUGUGACAGCGGCU	10.0256	6.8729	1.4587	0.1162	8.4869	7.2670	1.1679	0.4344
CUGUGCGUGUGACAGCGGCUGAU	7.3747	7.0044	1.0529	0.7069	8.2708	8.4087	0.9836	0.8424
CUGUGCGUGUGACAGCGGC	1.6688	1.2961	1.2875	0.3378	1.1267	0.7187	1.5678	**0.0487**
ACUGUGCGUGUGACAGCGGCUGA	1.8607	2.9434	0.6322	0.0560	1.1489	1.6681	0.6887	0.0707
ACUGUGCGUGUGACAGCGGCUG	0.3215	0.5923	0.5427	0.3052	NA	NA	NA	NA
ACUGUGCGUGUGACAGCGGCU	2.1925	1.9243	1.1393	0.7436	3.1856	2.3298	1.3673	0.1040
CUGUGCGUGUGACAGCGGCUGAA	7.1310	6.3103	1.1301	0.4938	8.3580	6.1532	1.3583	**0.0044**
CUGUGCGUGUGACAGCGGCUGAUA	0.7301	0.0524	13.9455	**0.0112**	1.2802	1.0870	1.1778	0.4821
CUGUGCGUGUGACAGCGGCUGAAA	5.6719	3.1524	1.7992	**0.0370**	8.2720	6.9212	1.1952	0.3214
CUGUGCGUGUGACAGCGGCUGAAAU	0.5124	0.0349	14.6796	**0.0182**	0.8841	0.6200	1.4259	0.1142
CUGUGCGUGUGACAGCGGCUGAAU	5.6741	4.9682	1.1421	0.4794	8.5123	6.5743	1.2948	**0.0383**
CUGUGCGUGUGACAGCGGCUGU	7.1091	8.1233	0.8751	0.3488	8.3845	7.7964	1.0754	0.5403
CUGUGCGUGUGACAGCGGCUGAUU	2.0013	2.1335	0.9380	0.8227	2.6846	2.4225	1.1082	0.5241
ACUGUGCGUGUGACAGCGGCUGU	1.8866	2.9019	0.6501	**0.0184**	2.7441	3.1914	0.8598	0.2046
CUGUGCGUGUGACAGCGGCUGUU	0.4066	0.1864	2.1810	0.1741	NA	NA	NA	NA
CUGUGCGUGUGACAGCGGCUGUAU	NA	NA	NA	NA	0.4698	0.5702	0.8238	0.6607

Underline, adding A, U or mixed nucleotides; *P*-value based on *t*-test; bold marked *P*-value < 0.05; AVG, average

Supplementary Table S3. Top 10 gene ontology (GO) terms for targets of commonly deregulated miRNA in ccRCC

GO id	Description	q-value	Occurrences in Sample	Occurrences in Genome
**miRNA-200c-3p**				
GO:0038093	Fc receptor signaling pathway	1.47E-13	25	219
GO:0002768	immune response-regulating cell surface receptor signaling pathway	1.06E-12	27	296
GO:0038095	Fc-epsilon receptor signaling pathway	2.86E-12	21	166
GO:0048011	neurotrophin TRK receptor signaling pathway	7.68E-12	25	274
GO:0038179	neurotrophin signaling pathway	7.92E-12	25	277
GO:0008543	fibroblast growth factor receptor signaling pathway	3.75E-10	19	170
GO:0044344	cellular response to fibroblast growth factor stimulus	1.08E-09	19	183
GO:0071774	response to fibroblast growth factor	1.08E-09	19	183
GO:0007173	epidermal growth factor receptor signaling pathway	1.52E-09	20	213
GO:0038127	ERBB signaling pathway	1.78E-09	20	216
**miRNA-362-5p**				
GO:0030897	HOPS complex	1.17E-05	5	12
GO:0006354	DNA-templated transcription, elongation	0.61636971	5	108
**miRNA-363-3p**				
GO:0000289	nuclear-transcribed mRNA poly(A) tail shortening	0.01642573	7	31
GO:0010608	posttranscriptional regulation of gene expression	0.15691157	17	298
GO:0061384	heart trabecula morphogenesis	0.15691157	4	12
GO:0014009	glial cell proliferation	0.15691157	4	11
GO:0002320	lymphoid progenitor cell differentiation	0.15691157	4	12
GO:0006417	regulation of translation	0.18950051	11	152
GO:0010942	positive regulation of cell death	0.18950051	15	268
GO:0000288	nuclear-transcribed mRNA catabolic process, deadenylation-dependent decay	0.18950051	7	58
GO:0030014	CCR4-NOT complex	0.23063786	4	15
GO:0031124	mRNA 3'-end processing	0.23606028	8	88
**miRNA-204-5p**				
GO:0007178	transmembrane receptor protein serine/threonine kinase signaling pathway	5.43E-05	21	218
GO:0002768	immune response-regulating cell surface receptor signaling pathway	6.31E-05	24	296
GO:0005126	cytokine receptor binding	2.06E-04	16	148
GO:0007179	transforming growth factor beta receptor signaling pathway	2.06E-04	16	150
GO:0043565	sequence-specific DNA binding	2.06E-04	21	255
GO:0002429	immune response-activating cell surface receptor signaling pathway	2.64E-04	17	175
GO:0000981	sequence-specific DNA binding RNA polymerase II transcription factor activity	3.03E-04	18	200
GO:0071559	response to transforming growth factor beta	6.04E-04	16	169

GO:0071560	cellular response to transforming growth factor beta stimulus	6.04E-04	16	169
GO:0090101	negative regulation of transmembrane receptor protein serine/threonine kinase signaling pathway	6.74E-04	11	78
**miRNA-21-5p**				
GO:0071214	cellular response to abiotic stimulus	1.54E-04	21	160
GO:0034142	toll-like receptor 4 signaling pathway	1.54E-04	17	107
GO:0019787	small conjugating protein ligase activity	1.54E-04	25	221
GO:0004842	ubiquitin-protein ligase activity	1.54E-04	25	209
GO:0016881	acid-amino acid ligase activity	1.88E-04	25	232
GO:0071260	cellular response to mechanical stimulus	1.88E-04	11	43
GO:0016569	covalent chromatin modification	1.88E-04	27	265
GO:0042326	negative regulation of phosphorylation	3.09E-04	24	223
GO:0016570	histone modification	3.30E-04	26	260
GO:0097285	cell-type specific apoptotic process	3.30E-04	22	194
**miRNA-224-5p**				
GO:0038093	Fc receptor signaling pathway	1.47E-07	16	219
GO:0002768	immune response-regulating cell surface receptor signaling pathway	7.18E-07	17	296
GO:0038179	neurotrophin signaling pathway	1.12E-05	15	277
GO:0048011	neurotrophin TRK receptor signaling pathway	1.12E-05	15	274
GO:0038095	Fc-epsilon receptor signaling pathway	1.55E-05	12	166
GO:0008543	fibroblast growth factor receptor signaling pathway	1.69E-05	12	170
GO:0044344	cellular response to fibroblast growth factor stimulus	2.91E-05	12	183
GO:0071774	response to fibroblast growth factor	2.91E-05	12	183
GO:0007173	epidermal growth factor receptor signaling pathway	1.21E-04	12	213
GO:0048017	inositol lipid-mediated signaling	1.21E-04	11	173
**miRNA-155-5p**				
GO:0019901	protein kinase binding	4.52E-07	41	276
GO:0071560	cellular response to transforming growth factor beta stimulus	8.10E-05	27	169
GO:0071559	response to transforming growth factor beta	8.10E-05	27	169
GO:0038093	Fc receptor signaling pathway	2.52E-04	30	219
GO:0007179	transforming growth factor beta receptor signaling pathway	2.52E-04	24	150
GO:0051169	nuclear transport	2.52E-04	35	282
GO:0010608	posttranscriptional regulation of gene expression	2.76E-04	36	298
GO:0038095	Fc-epsilon receptor signaling pathway	3.31E-04	25	166
GO:0038179	neurotrophin signaling pathway	3.31E-04	34	277
GO:0006913	nucleocytoplasmic transport	3.55E-04	34	279
**miRNA-210-3p**				
GO:0036294	cellular response to decreased oxygen levels	2.51E-04	8	70
GO:0071453	cellular response to oxygen levels	2.51E-04	8	72
GO:0071456	cellular response to hypoxia	2.51E-04	8	68
GO:1901992	positive regulation of mitotic cell cycle phase transition	0.00435409	5	26
GO:1901989	positive regulation of cell cycle phase transition	0.00435409	5	27
GO:0036293	response to decreased oxygen levels	0.00483169	8	118

GO:0070482	response to oxygen levels	0.004831<br>69	8	120
GO:0001666	response to hypoxia	0.004831<br>69	8	116
GO:0010639	negative regulation of organelle organization	0.004831<br>69	9	163
GO:0007059	chromosome segregation	0.006471<br>4	8	128

Supplementary Table S4. Primers used in the experimental procedures.

miRNA	primer sequence
universal reverse primer	CCAGTCTCAGGGTCCGAGGTATTC
Anchor RT primer	CGACTCGATCCAGTCTCAGGGTCCGAGGTATTCGATCGAGTCGCACTTTTTTTTTTTTVN
miR-200c-3p_F	TAATACTGCCGGGTAATGAT
miR-363-3p_F	AATTGCACGGTATCCATC
miR-362-5p_F	AATCCTTGGAACCTAGGT
miR-204-5p_F	TTCCCTTTGTCATCCTAT
miR-21-5p_F	CAACACCAGTCGATGGGC
miR-224-5p_F	TCAAGTCACTAGTGGTTC
miR-155-5p_F	TTAATGCTAATCGTGATA
miR-210-3p_F	CTGTGCGTGTGACAGCGG
miR-127-3p_F	CGGATCCGTCTGAGCTTG
miR-139-5p_F	TACAGTGCACGTGTCTCC
miR-424-5p_F	CAGCAGCAATTCATGTTTTG
miR-146b-5p_F	GAGAACTGAATTCCATAGGC
miR-183-5p_F	ATGGCACTGGTAGAATTC
miR-218-5p_F	TGTGCTTGATCTAACC
iso-miR-363-3p_F	TTGCACGGTATCCATCTGTA
iso-miR-224-5p_F	GTCACTAGTGGTTCCGTTTAG
U6_F	CAAATTCGTGAAGCGTTCC
TUT4_F	TGCTCAACAGGTGGCTGGTTCA
TUT4_R	GAGTTCTGTGGAAATGGCTGAGG
PAPD4_F	AGTTCGTCCGTTAGTGCTGGTG
PAPD4_R	GAGGGATGGAAGGATGGGTTCA
PAPD5_F	AGTGTCGTCCTCTTCTGCCACA
PAPD5_R	AACTGCCTGAGAGGACTCCAAG
DROSHA_F	GAAACTTCGCCACCTCCTAGCA
DROSHA_R	CTCCACCGTTACTTCTCGTCTC
DICER_F	ACTGCTGGATGTGGACCACACA
DICER_R	GGCTTTCCTCTTCTCAGCACTG
DGCR8_F	GGAAATCCGAGGTCTGCATCCT
DGCR8_R	GAGGCACCAAAAGGCTCACTTG
GAPDH_F	CATCAATGGAAATCCCATCAC
GAPDH_R	GGTTTTTCTAGACGGCAGGTC

F, forward primer; R, reverse primer; V, base A, G or C; N, any base
